# The effects of universal parental support programs: a systematic review and meta-analysis

**DOI:** 10.1186/s12889-026-28844-6

**Published:** 2026-09-28

**Authors:** Linus Haglund, Carl Lovén, Lars-Göran Öst, Agneta Herlitz, Pia Enebrink

**Affiliations:** 1 Department of Clinical Neurosciences, Karolinska Institutet, Stockholm, 171 77 Sweden; 2 Department of Psychology, Stockholm University, Stockholm, Sweden

**Keywords:** Children, Externalizing problems, Internalizing problems, Meta-analysis, Parenting, Universal parental support program

## Abstract

**Background:**

There are few current comprehensive meta-analyses and systematic reviews of the literature on universal parental support programs. The aim of this meta-analysis was therefore to examine the effects of universal parental support programs on children’s externalizing and internalizing problems, as well as on parental mental health, and to evaluate the possible importance of theoretical frameworks in achieving better outcomes.

**Methods:**

The meta-analysis included randomized controlled trials of universal parental support programs for parents to children and adolescents aged 2–17 years. A search in five databases was conducted from inception to December 2020 and updated in April 2024. The abstracts and subsequent full-text articles were independently reviewed by two pairs of authors, resulting in 31 original studies (36 articles) with a total of 7635 participants. To evaluate risk of bias, we used the Cochrane risk-of-bias tool for randomized trials.

**Results:**

Small positive short-term effects were obtained for the included universal parental support programs on parental ratings of children’s externalizing (*d* = 0.18: 95% CI 0.07–0.29) and internalizing problems (*d* = 0.16: 95% CI 0.03–0.30), parental stress (*d* = 0.22: 95% CI 0.04–0.40) and parental depression (*d* = 0.13: 95% CI 0.01–0.25). Subgroup analyses showed that significant effects on primary outcomes were only found when interventions were compared to waitlist, not to an active control group. The long-term between-group effects were positive but small and non-significant. Most of the programs were based on social learning theory, no differences emerged between programs with different theoretical frameworks.

**Conclusion:**

This meta-analysis indicates that universal parental support programs, when compared to waitlist only, may have a small positive impact on children’s externalizing and internalizing problems as well as on parental stress and depression in the short time frame.

**Supplementary Information:**

The online version contains supplementary material available at https://doi.org/10.1186/s12889-026-28844-6.

## Introduction

During the past two decades, psychological ill-health among children and adolescents has become more common. One of four children manifest behavioural problems, depression, anxiety or substance abuse [1]. Retrospective and prospective studies indicate that adult psychological problems may originate in childhood and adolescence [2], underscoring the importance of early interventions. Children’s psychological problems are usually divided into externalizing and internalizing problems. Externalizing problems are defined as behavioural problems, such as aggressive and defiant behaviours, outbursts of anger, impulsivity, and serious norm or rule violations, whereas internalizing problems include symptoms of depression and anxiety such as sadness, difficulties concentrating, worry, emotional and social avoidance [3].

Parental behaviours play an important role in the development or prevention of child externalizing and internalizing problems, together with other risk and protective factors (e.g., [4–7]). Meta-analyses and reviews suggest that some of the parental behaviours with a negative impact on externalizing or internalizing problems are child abuse and neglect, harsh criticism, conflicts with the child or with other family members, high parental interference and control of the child’s life, and too little warmth towards the child [4, 5, 8]. For externalizing problems, coercive family processes are considered one of the most relevant risk factors (e.g., [7, 9]). The theory of coercive family processes stipulates that the parent(s) and child inadvertently reinforce each other into dysfunctional interactions. These cycles can, for instance, be initiated by the child reacting with negativity, resistance, or anger to the parents’ prompts. The parents respond to the child’s externalizing behaviour with negativity and/or anger, and escalating requests. This may escalate until one part gives in, which negatively reinforces the other person’s behaviour [7]. Consequently, it is assumed that early externalizing behaviours in children can lead to harsh or over-controlling behaviours in parents, and that such parental behaviours may contribute to early externalizing behaviours in children. When parents consistently show a lack of ability handling conflicts, communicating clear boundaries, or creating positive interactions with the child, the child’s emotional and social development may be inhibited, the parent-child relationship may be impaired, and dysfunctional child emotional regulation strategies may develop [10, 11]. On the contrary, positive parenting, including warmth, giving attention, praise and functionally avoiding negative parent-child interactions, are related to improved child well-being [5]. For instance, positive parenting behaviours, such as showing warmth and understanding of the child’s emotions were associated with lower relational aggression, whereas negative/harsh, uncommitted, and over-controlling parenting behaviours were associated with more relational aggression in the child [8]. In addition, it has been suggested that parental validation of emotions and social expressions improves children’s understanding of emotions, emotional regulation ability, social expression, and general psychological health [11–13].

There are several established, evidence-based parental support programs aiming at intervening with externalizing and internalizing problems in children through parenting behaviours (see e.g., [14–16]). Most evaluations of these programs are conducted with parents at a selective, indicated or treatment level (e.g., [17, 18]). Selective programs target individuals with some externalizing or internalizing problems or risk factors, indicated and treatment programs are for families with children who have already developed various levels of clinical problems. Universal programs, on the other hand, are offered to all parents in a population regardless of risk level [19]. The prevention paradox states that with a universal approach more people who could have developed a problem can be reached, compared to a selective or indicated approach. Since the majority of those who later develop problems are from low-risk groups, universal interventions will have an opportunity to reach more of those individuals who without an intervention will develop the problem [20]. Depending on how effective the universal intervention is it can have an important effect on reducing the risk for future problems and at the same time decrease the risk for future individual suffering and societal costs [21].

A systematic review examining the aggregated evidence in the literature of universal programs indicated that these programs seem to have a positive, small effect on children’s general mental health, but more uncertain effects on externalizing and internalizing problems [21]. Han and colleagues [22] conducted a meta-review of RCTs of parenting interventions, finding effects for universal parenting interventions on delaying child alcohol and cannabis initiation. In this narrative review, the authors found mixed evidence for the effects on reduced child externalizing or internalizing problems. A key motivation for conducting a new meta-analysis is that this can provide a precise pooled effects estimate and systematically examine variation across studies. A meta-analysis of parenting programs based on cognitive behavioural therapy (CBT) for children aged 2–9 years comparing programs at different prevention/intervention levels [17], indicated that universal programs (*n* = 8) had a small non-significant effect size, whereas selective (*n* = 50), indicated (*n* = 50) and treatment programs (*n* = 45) had small to medium significant effect sizes on children’s externalizing problems [17]. The meta-analysis by Leijten and colleagues only included CBT-based programs, children within a certain age-frame and externalizing problems as outcome, which points to a need to complement these with studies targeting other age groups, theoretically diverse programs and other outcomes.

Parental support programs at the selective and indicated level seem to lead to improvements in parents’ psychological ill-health, such as depression, anxiety, stress-related symptoms [14, 23]. A child developing externalizing problems may contribute to a negative impact on parental stress [24], which in turn might have a negative impact on the child’s continued problems. For instance, a decrease in depressive symptoms was seen in divorced mothers of boys who underwent parental support programs, which was mediated by a reduction in the boys’ externalizing problems [25]. That is, parents’ psychological health can be both an antecedent and a consequence of children’s externalizing problems, and children’s externalizing problems can be an antecedent and a consequence of parents’ stress [26], why program effects on parental mental health outcomes would be important to synthesize at the universal level. Improvements in parental mental health could be expected also in universal parenting programs as these interventions often strengthen general protective factors, such as parenting self-efficacy, parenting practices and stress management, which would be of benefit for parental well-being regardless of child difficulties.

The effect of a parental support program may depend on factors related to the treatment approach, theoretical framework and participant characteristics [27]. Different theories have been used as a basis for various parental support programs. Whether a theoretical framework improves the effectiveness has not been evaluated in a meta-analysis for universal parental support programs. Such knowledge would increase the possibility of optimizing parental support programs. Most universal programs build on social learning theory (CBT), emotion theory, attachment theory or psychodynamic theory, thus providing a variety of theoretical frameworks of possible relative importance for different populations and outcomes. Whereas there are few systematic reviews and meta-analyses of emotion-oriented parenting programs, evaluations of universal CBT-based parenting interventions suggest positive, although non-significant, effects on child outcomes [17]. Few syntheses have focused specifically on the universal prevention context and integrated emotion-focused and CBT-based parenting programs.

To understand who might benefit more from universal parenting interventions and which treatment or study characteristics are associated with improved outcomes, we included common study descriptives from earlier systematic reviews (i.e., percent mothers, percent boys/girls, child age) as well as various treatment characteristics (i.e., number of sessions, treatment time, treatment format), and evaluated whether methodological aspects such as the number of participants in the study, as a proxy for statistical power, and the handling of missing data through completer vs. intention-to-treat-analyses influenced the outcomes. All moderators were decided a priori.

The purpose of the current meta-analysis is to fill the mentioned research gaps by providing a comprehensive meta-analysis and systematic review of the literature on universal parental support programs. The aims were to: (1) evaluate the effect of universal parental support programs on externalizing and internalizing problems among children and adolescents aged 2–17 years in the short and long term; (2) evaluate the effect of universal parental support programs on parents’ stress, depression and anxiety in the short and long term; (3) examine if the effect of parental support programs on externalizing and internalizing problems in children is moderated by the number of participants in the studies, percent of mothers, percent of boys, child’s age, number of program sessions, program treatment time, program format (group or individual) or the statistical analysis employed (intent-to-treat or completer-analysis); (4) examine the possible importance of various theoretical frameworks for improved short and long term effects. Based on the earlier review and meta-analysis [17, 21], we predicted that the short and long-term effects would be small and positive, that child age and gender would moderate outcome, and that programs based on social learning theory/CBT would be related to improved outcomes.

## Method

This systematic review follows the PRISMA 2020 guidelines [28]. It targets randomized controlled trials of universal parental support programs and was designed according to the following PICOS:

### Population

Parents of children and adolescents aged 2–17 from the general population. Parents were defined as primary caregiver responsible for taking care of the child. Studies targeting foster families were excluded, as this group was assumed to be at higher risk for developing problems than the general population. We also excluded parenting programs of very young children, between 0 and 2 years, since these may differ from parenting interventions of older children.

### Intervention

We only included interventions that were universally structured. Studies targeting risk or vulnerable groups were excluded, for example studies on parents with low-income, refugees, children at risk for developing ADHD, externalizing problems, families in vulnerable areas, or divorced parents. Only programs based on a psychological theory were included. For inclusion, a therapist contact was required in the intervention, either face-to-face, via the internet or by phone. We only included parental support programs targeting parents and therefore all studies with interventions involving children, teachers, or persons other than the primary caregivers were excluded.

### Comparison group

An active control group or no treatment control group (passive control group, which meant for example receiving a short information sheet or nothing), treatment as usual, or wait list group was accepted. Studies without a comparison group were excluded.

### Outcome

The primary outcomes were externalizing or internalizing problems in children, measured through parental or teacher assessment, child assessment or observations. Studies that did not evaluate externalizing or internalizing problems in children or only presented one merged total score for these outcomes were excluded. The secondary outcome was parental mental health in terms of stress, depression, or anxiety, measured by self-assessments. Only studies using continuous measures were included. Studies that did not report data enabling the calculation of effect size, post- or follow-up assessments were excluded.

### Study design

We only included randomized controlled trials or cluster randomized controlled trials with at least ten participants in each intervention and comparison group, published in English-language journals. Both cluster-randomized and individually randomized controlled trials were included, as they represent rigorous experimental designs that minimize bias and allow for causal inference. Dissertations and secondary analyses were excluded.

### Procedure

#### Literature search

**Fig. 1:**
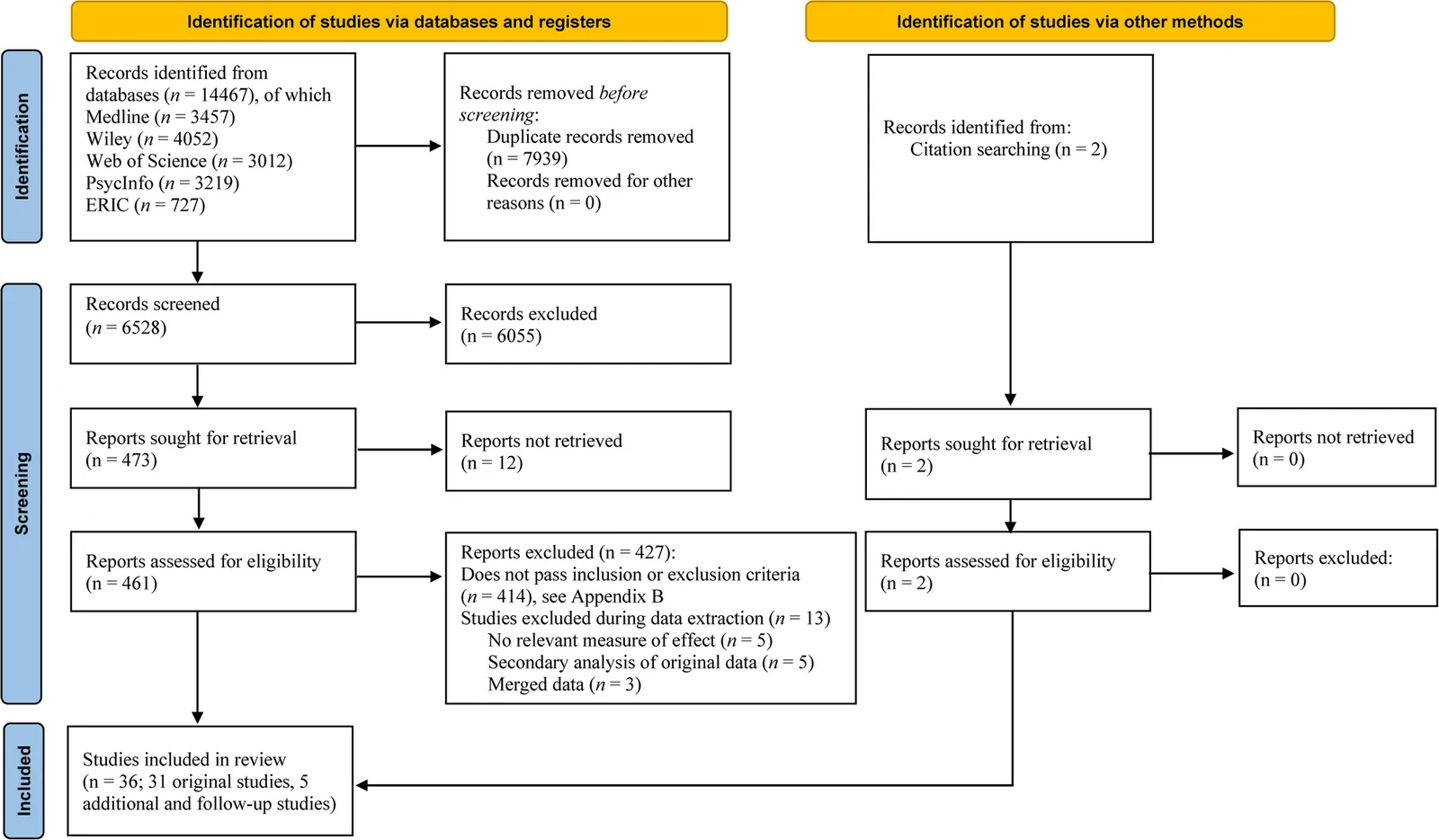
PRISMA 2020 flow diagram for systematic reviews. Adapted from Page MJ, et al. [28]; CC BY 4.0.

At two occasions, a systematic literature data base search was performed by librarians at the Karolinska Institute University Library: 22 December 2020 and 22 April 2024. Five databases were used: Medline (Ovid), Cochrane Central Register of Controlled Trials (Wiley), Web of Science Core Collection, PsycInfo (Ovid) and ERIC (ProQuest). Unpublished and non-peer-reviewed studies were not included in the search. Keywords used in the search were, for example, “universal parenting program”, “child behaviour”, “behaviour problems”, “parenting”, “randomized controlled trial”, resulting in 6528 abstracts. The abstracts and the subsequent full-text articles were independently reviewed by two pairs of authors (LH, CL, PE, AH), and any disagreements were solved in discussion to reach consensus, sometimes with the help of the last author (PE). Abstract screening was conducted using the Rayyan systematic review tool [29]. A total of 461 studies were reviewed in full text, independent of each other’s assessment. After the full-text review, another two suitable studies were identified through review of previous systematic reviews in the field, see Fig. 1 for details. Appendix A presents the search strategies, Appendix B the inclusion/exclusion criteria, and Appendix C the reasons for exclusion during the full-text review (see Supplementary Material 1).

#### Data extraction

Each data extraction was double-checked by one of the other authors. During the extraction process there were continuous meetings and e-mails to discuss ambiguities with data until consensus was reached. For example, how to categorize the theory base was discussed. A decision was made to examine the parenting program’s website to determine its theoretical basis if this was not explained in the study or its sources. Some programs were based on several theories, for example both social learning theory and emotion theory. Both program orientations were then noted. When the rating scale Eyberg Child Behavior Inventory was used as a measure, the intensity scale was included [30]. We aimed to include common parent mental health issues, such as stress, depression and anxiety.

For each included article, we extracted characteristics of the study, study participants, the interventions, instruments used, means and standard deviations. In three cases, where mean or standard deviation for the primary outcomes were not reported, the reported effect size was extracted instead.

#### Moderators

Moderators were chosen after evaluation of common moderators presented and analysed in the literature. The moderators were: (1) total number of participants in the study (2), percentage of mothers (3), percentage of boys (4), average age of the children (5), number of treatment sessions (6), hours in treatment.

#### Classification of programs

A review of the theoretical basis of each parental support program was performed. The programs were classified according to the usual types of programs in the literature.

### Evaluation of risk-of-bias

The Cochrane risk-of-bias tool for randomized trials was used, the original version for individually randomized trials and a custom version for the cluster randomized trials [31]. For articles included in the first search, six articles were evaluated by the first and fifth author individually and thereafter compared and discussed to reach consensus. After this, the first and fifth author evaluated twelve or thirteen different studies each. All five articles included in the second search were first individually evaluated and thereafter discussed by fourth and fifth author until consensus was reached.

### Statistical analysis

Between-group effect size (ES) was calculated as Cohen’s *d* (M_intervention_ – M_control_)/SD_pooled,_ separately for post- and follow-up assessment [32], where values around 0.2 indicate a small effect, around 0.5 a medium effect, and 0.8 or higher a large effect. Within-group ES was calculated as (M_pre_ – M_post_)/SD_pre_ according to a recommendation by Lakens [33], since there is good reason to assume that the interventions influence not only the means but also the standard deviations. The mean ES was computed by weighting each ES by the inverse of its variance. We used intent-to-treat (ITT) data when a study provided those, otherwise completer data were used.

Before pooling, the between group effect sizes were screened for statistical outliers, defined as being outside M ± 2SD. There were five outliers at the post-assessment and one at follow-up, making a total of six (4.8%) of the 126 between group effect sizes in this meta-analysis. Three of the outliers were within the interval when combined with a second study-measure of the same outcome. For the other ESs, *winsorizing* [34] was used by reducing outliers to the value of M+2SD. The *Comprehensive Meta-Analysis v.4* (CMA; [35]) was used for all analyses. A random effects model was used since it cannot be assumed that the ESs come from the same population.

Heterogeneity among ESs was assessed with the *Q*-statistic and the prediction interval (the true effect size in 95% of all comparable populations will fall within this interval; [35]), and publication bias with funnel plots, Egger’s regression intercept [36], and Duval and Tweedie’s [37] trim and fill method. Moderator analyses of categorical variables were done with subgroup analysis using the mixed effect model and of continuous variables with meta-regression using the random effects model.

#### Outcomes

Four different outcomes were evaluated: Primary outcomes were children’s externalizing and internalizing problems, and secondary outcomes were parental stress, anxiety and depression. Analyses were performed separately for each outcome and were separated for different informants (parent, teacher, or child). Analyses were performed for post measurements and follow-up measurements, separately for within group and between group measurements. Post measurement, i.e., short-term follow-up, was defined as the first assessment after the intervention had been completed, and long-term follow-up as the last assessment results reported. For studies that presented several follow-up assessments, data from the longest follow-up were used. In within group analyses, a pre/post-correlation of 0.5 was used. Sensitivity analysis was performed.

Although a meta-analysis technically can be conducted with two effect sizes [38], we required at least three studies per outcome. This decision was made to improve stability and interpretability of the pooled effect size and reduce the risk that results are disproportionately influenced by single studies.

#### Moderator analysis

Meta-regression analyses were conducted for the potential continuous moderator variables when significant heterogeneity was discovered. Subgroup analyses were used for categorical variables. To control type 1 errors, Holm-Bonferroni correction was used to correct the *p*-values [39].

## Results

### Studies

The flowchart (Fig. 1) summarizes the search results. After the screening of 6528 records, 461 full reports were assessed for eligibility. Of these, 427 reports were excluded due to inclusion or exclusion criteria (*n* = 414), or during the data extraction process (*n* = 13) as there were no relevant rating scales, follow-up data had already been included or data was not reported in a way that could be used for the meta-analysis, leaving 34 studies. As two additional records were identified from citation searching, there were in total 36 studies [40–75] available for the meta-analysis (see Flowchart, Fig. 1). One of these 36 studies presented other outcomes from the same original trial [40] and four studies were follow-up studies of included studies [41–44]. Since they included the same participants, the follow-up studies were not analysed as separate studies unless they included new data. The most common measures of parental mental health in the included studies targeted stress (*n* = 9) and depression (*n* = 6). Too few studies included parental anxiety as an outcome for it to be considered in the analyses. Four of the included studies had an overall measure of parents’ mental health, but all measured different aspects, which is why global parental mental health was not included as an outcome. The parental outcomes analysed were stress and depression.

### Background data

The meta-analysis included data from 31 original randomized or cluster randomized controlled trials on universal parental support programs, four follow-up studies and one study with complementary outcome data, see Table 1. The number of participants in the studies ranged from 31 to 1675, with a total of 7708 participants. When aggregated across all included studies, the mean age of the children was approximately 7 years. There were overall slightly more boys (53.5%) than girls.

**Table 1:** Descriptives of the included studies and demographic characteristics of the participants

Author(s)	Year	Country	Comparison	Randomization	Number of participants	% Mothers	% Boys	Age range of children, years	Age (M) of children	Follow up (year)^1^	Total risk of bias
Altafim	2019	Brazil	WL	I	81	100	58	3–8	4.2	0.3^2^	High
Amador Buenabad	2020	Mexico	CG	C	207	83	57	2–5th grade	8.6	–	Some
Bodenmann	2008	Switzerland	CG	I	200	50	55	2–12	6.6	1	High
Burke	2012	Australia	WL	I	180	90	54	10–14	11.9	–	High
Burkhardt	2024	Switzerland	WL	I	141	88	57	3–6	5.0	0.5	Some
Chu	2015	New Zealand	WL	I	144	100	59	12–15	11.9	0.5	Some
Dadds	2008	Australia	CG	C	734	–	53	3–6	Most 4 to 6y	0.6	Some
Foskolos	2023	Greece	CG	I	123	85	54	2–12	5.5	0.5	High
Grolnick	2023	USA	WL	I	57	95	40	8–12	9.9	–	Some
Hahlweg	2008	Germany	WL	I	63	91	50	3–6	3.6	–	Some
Hahlweg	2010	Germany	CG	C	282	52	51	3–6	4.5	2	Some
Havighurst	2010	Australia	CG	C	327	96	52	4–6	4.7	0.5	Some
Kehoe^a^	2014	Australia	CG	C	225	89	49	10–13	12.0	–	Some
Leung	2017	Hong Kong	WL	C	173	87	52	2–3	2.5	0.3^2^	Some
Leung	2023	Hong Kong	WL	C	242	80	50	–	Preschool	–	Some
Lösel^b^	2009	Germany	CG	C	290	96	50	–	4.7	5	Some
MacKenzie	2000	USA	CG	I	31	90	49	3–5	4.3	0.1	Some
Malti^c^	2011	Switzerland	CG	C	1675	–	52	1st grade	7.5	6	Some
Matsumoto	2007	Australia	WL	I	50	96	54	2–10	4.9	0.3^2^	Some
Nitsch	2015	Ireland	WL	I	109	85	39	10–16	12.3	0.5^2^	Some
Ozturk	2019	Italy	WL	I	44	75	66	–	15.0	–	Some
Qui	2022	China	WL	I	89	100	58	3–6	4.9	–	Some
Reedtz^d^	2011	Norway	CG	I	186	100	59	2–8	3.9	4	Some
Rincon	2018	Chile	WL	C	332	87	53	3–4	3.7	–	Some
Sampaio	2015	Sweden	CG	C	759	59	–	2–5	–	1.5	High
Schilling	2020	USA	WL	I	100	93	57	2–6	2.6	–	Some
Smith	2018	USA	CG	I	343	100	54	4–12	7.8	0.5	High
Sumargi	2015	Australia	WL	I	143	94	50	2–12	6.3	0.5^2^	Some
Webster-Stratton	1982	USA	WL	I	35	100	66	3–5	3.9	0.3^2^	Some
Wilson	2012	Australia	WL	C.	128	92	52	4–6	4.2	–	Some
Yap^e^	2018	Australia	CG	I	359	86	55	12–15	13.7	1	Some

A dash means that no data were reported, ^1^Longest study follow-up time included, ^2^Follow-up data for intervention group, not control group, a = Additional study on other outcomes by Havighurst et al. [40], b = Follow-up by Lösel et al. (2013), c = Follow-up by Averdijk et al. [41], d = Follow-up by Reedtz & Klest (2016), e = Follow-up by Yap et al. [44]; follow-up studies are included in this meta-analysis and have the same descriptives as their original study, except for a change in age of years (*M*) of children; Comparison: WL = wait list, CG = control group; Randomization: I = individual, C. = cluster; Follow up assessment is in years after post measurement

Follow-up data were collected on average close to one and a half year after post measurement (*M* = 1.3 years, *SD* = 1.7). The shortest follow-up period was one month and the longest six years. Six of the original studies also included uncontrolled 3 to 6-month follow-up data of the parent group, i.e., without data from the control group. These studies were included in the within-group analyses.

Publications of the included studies extend over 42 years (1982–2024; 24 years if excluding Webster-Stratton, 1982; 58 [67]). Studies were conducted in Europe (*n* = 11), Australia/New Zealand (*n* = 9), North America (*n* = 5), Asia (*n* = 3) and South America (*n* = 3). The programs had an average duration of seven weeks (*M* = 7.25, *SD* = 3.44) and seven sessions (*M* = 7.15, *SD* = 3.30). Total time in the programs for participants in the intervention groups was more than twelve hours (*M* = 12.45, *SD* = 7.02, Mdn = 12, range = 3–40). Twenty-six studies used group-based program, four studies had individual training, and one study had both group and individual training.

Table 2 presents the names and theoretical classifications of the parental support programs. All programs were based on a manual. The 31 included studies evaluated 17 different programs. As can be seen in Table 2, thirteen different programs were classified as CBT-based (evaluated in 23 studies; [45–67]). One program was classified as emotion-based and was evaluated in five studies [68–72]. There were also programs classified as based on self-determination theory, attachment theory or risk/factor-based program, evaluated in one study each [73–75]. For purpose of analysis, these three were merged into a category called “other”.

**Table 2:** Classification and name of parental support program

Author(s)	Year	Classification	Program	Treatment time^c^	Sessions	Treatment format
Altafim	2019	CBT	ACT Raising Safe Kids Program	16	8	G
Amador Buenabad	2020	CBT	CAPAS-Mx: Raising children with Love	18	12	G
Bodenmann	2008	CBT	Triple P	12	8	G
Burke	2012	CBT plus^a^	ABCD Parenting young adolescent program	12	6	G
Burkhardt	2024	Emotion-based	Tuning in to Kids	–	6	G (online)
Chu	2015	CBT	Triple P	17.5	10.5	G
Dadds	2008	CBT	REACH for RESILIENCE	–	6	G
Foskolos	2023	CBT	Triple P (seminar series)	4.5	3	G
Grolnick	2021	Other^b^	Parent Check-In	3	2	I
Hahlweg	2008	CBT	Triple P	11.8	7	I
Hahlweg	2010	CBT	Triple P	9	8	G
Havighurst	2010	Emotion-based	Tuning in to Kids	16	8	G
Kehoe	2014	Emotion-based	Tuning in to Teens	12	6	G
Leung	2017	CBT	HOPE-20	40	20	G
Leung	2023	CBT plus	The Happy Parenting: Round-the-Clock Parenting Program	16	8	G
Lösel	2009	CBT	EFFEKT	10	5	G
MacKenzie	2000	CBT	The Computer-Assisted Parenting Program	7.5	5	I (online)
Malti	2011	CBT	Triple P	13.3	4	G
Matsumoto	2007	CBT	Triple P	11.5	8	G
Nitsch	2015	CBT plus	Parents Plus Adolescents Programme	–	8	G
Ozturk	2019	Other	Connect	15	10	G
Qui	2022	Emotion-based	Tuning in to Kids	12	6	G
Reedtz	2011	CBT plus	The Incredible Years	12	6	G
Rincon	2018	CBT	Day by Day program	16	6	G
Sampaio	2015	CBT	Triple P	5.8	7	G + I
Schilling	2020	CBT	PriCARE	9	6	G
Smith	2018	CBT	Triple P	20	10	G
Sumargi	2014	CBT	Triple P	4.5	3	G
Webster-Stratton	1982	CBT plus	The Incredible Years	8	4	G
Wilson	2012	Emotion-based	Tuning in to Kids	12	6	G
Yap	2018	Other	Partners in Parenting program	4.5	9	I (online)

A dash means that no data were reported, a = CBT plus stands for programs that are based on CBT but also contain elements from other theories, b = other includes a self-determination based program, an attachment based program and a program based on risk- and protection factors, c = maximum time spent in the program for parents in the intervention group, including booster sessions and optional sessions, Treatment format: G = group, I = individual. Online = computer-based program with therapist feedback

Thus, social learning theory and CBT-based programs, provided by far the most common theoretical basis. This reduced the possibility of conducting an analysis on type of program. For an exploratory, hypothesis-generating purpose, a division was made between the programs that contained pure CBT and those that contain both CBT plus elements based on other theories. Of the 23 studies that were originally coded as CBT, 18 were judged to be based on programs with pure CBT, while five of the studies were based on programs that also contained elements from other theories. The Incredible Years (two studies; [61, 67]) were identified to contain, in addition to CBT, elements of attachment theory, ABCD Parenting young adolescent program (one study; [48]) relies heavily on acceptance-based strategies, The Happy Parenting: Round-the-Clock Parenting Program (one study, [55]) on social learning theory and emotion coaching, and Parents Plus Adolescents Program (one study; [60]) contains elements of psychodynamic theory. The most used programs were the Triple P (*n* = 10) and Tuning in to Kids/Teens (*n* = 5).

### Risk of bias

In line with the Cochrane Risk of Bias tool for randomized trials [31], the studies were coded as “low”, “some concerns”, and “high” risk of bias in each of the five domains and given a total risk of bias evaluation based on these assessments. The presented risk of bias estimates are based on the original study, i.e., including 31 studies. The overall risk estimate did not differ for the additional or follow-up studies (*n* = 5), although there were a few minor differences at item level e.g. due to different measures or data analyses than in the original study. In domain 1 (risk of bias arising from the randomization process), the majority of studies [27] had a low risk of bias (2 some, 2 high). In domain 2 (risk of bias due to deviations from the intended interventions), 25 studies had a low risk of bias (5 some, 1 high). In domain 3 (risk of bias due to missing outcome data), 19 studies had a low risk of bias (10 some, 2 high), In domain 4 (risk of bias in measurement of the outcome), 3 had low risk of bias (23 some, 5 high risk). In domain 5 (risk of bias in selection of the reported result), 5 studies had low risk of bias (26 some risk). The higher bias-ratings in domains 4 and 5 were related to non-blinding procedures and that parents were main informants of outcomes, but also to differences in evaluation time points for the active and control groups (one study) and high attrition without analysis (one study) as well as that few studies had registered study protocols or had detailed information about analyses. Most studies (25; 80.6%) had a total risk of bias that indicated “some” biases. There were six studies (19.4%) with “high” total risk of bias.

### Objective 1: Primary outcomes – child externalizing and internalizing behaviours

**Fig. 2:**
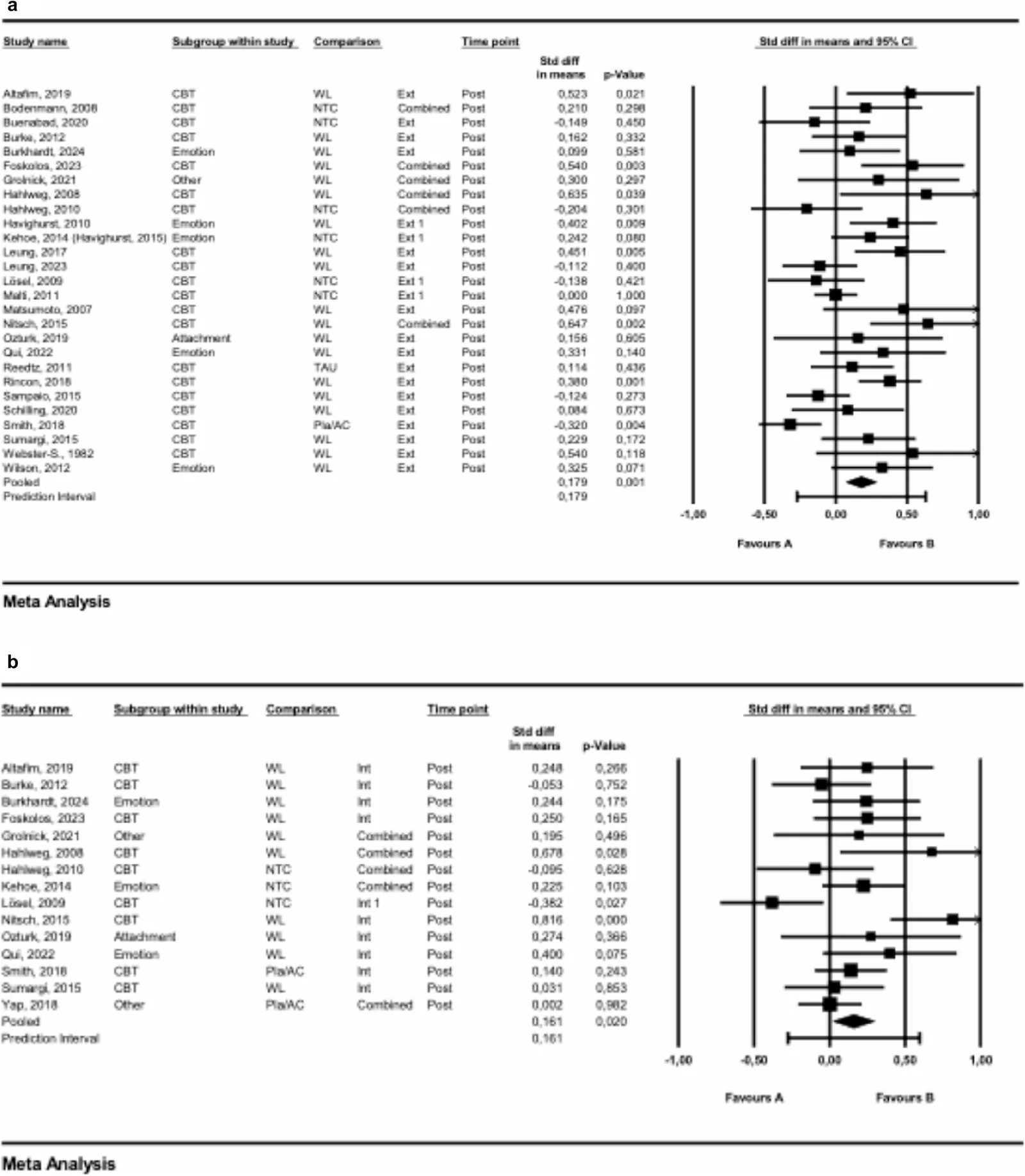
**a** Forest plot of standardized mean differences in parental ratings of externalizing behaviours at post assessment across included studies. **b** Forest plot of standardized mean differences in parental ratings of internalizing behaviours at post assessment across included studies

Table 3 shows effect sizes for the outcomes at post and follow-up assessments, comparing the intervention to all control groups together, and for the primary outcomes also separately to waitlist and other control groups. Forest plots of the main outcomes, i.e., parent-rated child externalizing and internalizing behaviours, are presented in Figs. 2a-b. We found small significant effect sizes for parent-rated child externalizing problems (*d* = 0.18; 95% CI 0.07–0.29) and parent-rated child internalizing problems (*d* = 0.16; 95% CI 0.03–0.30). Teacher and child evaluations of child externalizing behaviours were not significant. The long-term between-groups effects between parenting support programs and control groups are also shown in Table 3. There were no significant differences between the groups at the follow-up. Table 4 presents within group effect sizes for the parent support programs, from pre- to post and follow-up assessments. From pre- to post assessment there was a significant reduction with a small effect size in parent-rated child externalizing problems (*d* = 0.36: 95% CI 0.25–0.47), parent-rated child internalizing problems (*d* = 0.34: 95% CI 0.23–0.45) and child-rated child externalizing problems (*d* = 0.21: 95% CI 0.11–0.31). From pre- to follow-up assessment, there was a significant reduction with small to medium effect sizes in parent-rated child externalizing problems (*d* = 0.48: 95% CI 0.35–0.61), parent-rated child internalizing problems (*d* = 0.44: 95% CI 0.30–0.58), and teacher-rated externalizing problems (*d* = 0.33: 95% CI 0.16–0.49).

**Table 3:** Between group effect sizes at post and follow-up, divided on raters, outcomes and control groups

Time point/Outcome	*k*	*d*	95% CI	*z*-value	Q-value	95% PI	*I*^*2*^ (%)
**Post**							
*Parent ratings*							
Externalizing	27	0.18	0.07; 0.29	3.31^b^	70.9^c^	-0.27; 0.63	63.5
vs WLC	19	0.28	0.16; 0.40	4.70^c^	35.0^c^	-0.11; 0.67	48.6
vs Control	8	-0.03	-0.18; 0.11	-0.45	14.9^a^	-0.43; 0.37	52.9
Internalizing	15	0.16	0.03; 0.30	2.32^a^	30.3^b^	-0.28; 0.60	53.8
vs WLC	10	0.28	0.11; 0.45	3.19^b^	14.8	-0.16; 0.71	39.0
vs Control	5	0.003	-0.18; 0.19	0.03	9.0	-0.58; 0.58	55.4
Parental stress	9	0.22	0.04; 0.40	2.44^a^	17.6^a^	-0.27; 0.71	54.6
Parental depression	6	0.13	0.01; 0.25	2.15^a^	5.5	-0.08; 0.35	8.5
*Teacher ratings*							
Externalizing	3	0.13	-0.39; 0.65	0.48	43.2^c^	-6.47; 6.72	95.4
*Child ratings*							
Externalizing	3	0.05	-0.08; 0.17	0.72	1.3	—	0.0
**Follow-up**							
*Parent ratings*							
Externalizing	11	0.13	-0.03; 0.29	1.64	26.3^b^	-0.35; 0.61	62.0
vs WLC	4	0.18	-0.07; 0.43	1.43	8.4^a^	-0.85; 1.22	64.2
vs Control	7	0.10	-0.12; 0.32	0.88	17.4^b^	-0.56; 0.75	65.5
Internalizing	6	0.07	-0.12; 0.27	0.72	11.1^a^	-0.50; 0.64	54.7
vs WLC	2	-	-	-	-	-	-
vs Control	4	0.06	-0.24; 0.35	0.40	10.0	-1.19; 1.31	69.9
Parental stress	3	0.03	-0.11; 0.17	0.40	1.4	—	0.0
*Teacher ratings*							
Externalizing	5	0.14	-0.03; 0.30	1.59	7.7	-0.35; 0.61	48.2
*Child ratings*							
Externalizing	3	0.32	-0.19; 0.83	1.22	10.26^a^	-5.73; 6.36	80.5

k = number of studies; d = Cohen’s d; CI = confidence interval; z = test of significance for d; a = *p* < 0.05; b = *p* < 0.01; c = *p* < 0.001; Q = statistical test of heterogeneity; PI = prediction interval; I2 = proportion of the observed variance that reflects variance in true effects. WLC = waitlist control; Control group includes NTC = no treatment control (passive control group), Placebo-attention control or Treatment as Usual (TAU; active control groups) which were not analyzed separately due to too few studies

### Objective 2: secondary outcomes – parental stress, depression, and anxiety

Parental anxiety at post measurement was not analyzed due to having less than three studies. Forest plots for secondary outcomes are available in the Supplementary Material 2 (Figures S1–S9). We found small significant effect sizes for parent-rated parental stress (*d* = 0.22; 95% CI 0.04–0.40), and a very small significant effect for parent-rated parental depression (*d* = 0.13; 95% 0.01–0.25). There were no significant differences between the groups at the follow-up (Table 3). Table 4 presents within group effect sizes for the parent support programs, from pre- to post and follow-up assessments. There was a significant reduction with a small effect size in parent-rated parental stress (pre- to post assessment *d* = 0.30: 95% CI 0.14–0.45, pre- to follow-up assessment *d* = 0.35: 95% CI 0.13–0.56)). A very small effect size was found for reduction in parent-rated parental depression which showed significance over the pre- to post assessment but not over the follow-up (*d* = 0.13: 95% CI 0.04–0.21).

**Table 4:** Within group effect sizes at post and follow-up, divided on raters and outcomes

Time point/Outcome	*k*	*d*	95% CI	z-value	Q-value	95% PI	*I*^*2*^ (%)
**Post**							
*Parent ratings*							
Externalizing	25	0.36	0.25; 0.47	6.47^c^	131.3^c^	-0.15; 0.88	81.7
Internalizing	13	0.34	0.23; 0.45	6.19^c^	25.7^a^	0.01; 0.68	53.2
Parental stress	9	0.30	0.14; 0.45	3.67^c^	32.9^c^	-0.20; 0.79	75.7
Parental depression	6	0.13	0.04; 0.21	2.98^b^	5.8	-0.04; 0.29	14.0
*Teacher ratings*							
Externalizing	3	0.10	0.02; 0.19	2.36^a^	2.4	-0.57; 0.78	16.3
*Child ratings*							
Externalizing	3	0.21	0.11; 0.31	4.19^c^	2.2	-0.54; 0.97	9.4
**Follow-up**							
*Parent ratings*							
Externalizing	15	0.48	0.35; 0.61	7.39^c^	55.1^c^	0.01; 0.95	74.6
Internalizing	7	0.44	0.30; 0.58	6.18^c^	12.2^c^	0.06; 0.82	50.7
Parental stress	6	0.35	0.13; 0.56	3.18^c^	22.4^c^	-0.34; 1.03	77.7
Parental depression	3	0.15	-0.10; 0.41	1.18	5.2	-2.61; 2.92	61.6
*Teacher ratings*							
Externalizing	4	0.33	0.16; 0.49	3.96 ^c^	11.4^b^	-0.37; 1.02	73.7

*k* = number of studies; *d* = Cohen’s *d*; CI = confidence interval; *z* = test of significance for *d*; a = *p* < 0.05; b = *p* < 0.01; c = *p* < 0.001; *Q* = statistical test of heterogeneity; *I*^2^ = level of heterogeneity

### Publication bias

Egger’s test for the regression intercept indicated publication bias for parent-rated externalizing behaviours (*t* = 2.17, *p* = 0.0137) due to asymmetry. Duval and Tweedie’s trim and fill method suggested trimming 6 studies, which would bring the *d* down to 0.10. No publication biases were indicated for the other outcomes.

### Objective 3: moderator analyses

#### Meta-regression of continuous variables

Most of the previous analyses showed significant medium to high heterogeneity, especially for the primary outcomes. Therefore, six potential continuous moderators were examined. The proportion of the studies that had information on these variables was on average 94.2% (range 90.3–100%). Meta-regression analyses were performed for the moderators on parent-rated externalizing and internalizing problems, see Table 5. One moderator, number of participants in the study was a significant negative moderator for child externalizing (*n* = 27, Coefficient = -0.0007, *p* = 0.0039) and internalizing problems (*n* = 15, Coefficient = -0.0014, *p* = 0.02), i.e., studies with higher number of participants were associated with a lower ES on externalizing problems. When the Holm-Bonferroni correction was applied, this moderator was still significant for externalizing problems but not for internalizing problems.

**Table 5:** Meta-regression analysis of parent ratings of continuous variables at post-assessment divided on outcomes

Variable	*k*	Coefficient	z-value	*p*-value
*Externalizing*				
No. of participants	27	-0.0007	-2.89	0.0039^a^
Percent mothers	25	0.0050	1.14	0.25
Percent boys	26	-0.0059	-0.53	0.60
Children’s mean age	25	-0.0099	-0.55	0.58
Number of sessions	26	-0.039	-1.58	0.11
Hours of treatment	26	0.0053	0.67	0.50
*Internalizing*				
No. of participants	15	-0.0014	-2.31	0.02
Percent mothers	14	-0.0085	-0.73	0.47
Percent boys	14	0.010	0.83	0.41
Children’s mean age	15	0.0073	0.37	0.71
Number of sessions	15	0.0149	0.48	0.63
Hours of treatment	14	0.0208	1.59	0.11

*k* = number of studies. ^a^  = significant also when Holm-Bonferroni correction was applied

#### Subgroup analyses

Differences in between-group effect sizes at post and at follow-up assessment for the different types of comparison groups, the wait list and no treatment control group, were evaluated for the externalizing and internalizing problems estimated by parents. Some differences were found, see Table 3. In studies where the intervention group was compared with wait list, a small effect size was seen for externalizing (*d* = 0.28: 95% CI 0.16–0.40) and internalizing (*d* = 0.28: 95% CI 0.11–0.45) problems. However, in studies comparing the intervention group with more active control groups (placebo, active control, treatment as usual), no significant difference was seen between groups. These control groups had less than three studies of each type, which is why they were analysed together. Even though effect sizes differed, there were no statistically significant differences in between group effect sizes for type of statistical analysis (ITT or completers), or treatment format (group or individual), see Table 6.

**Table 6:** Subgroup analysis of categorical variables for parent-rated problems at post-assessment, including all control groups

Variable	*k*	*d*	95% CI	Qb	*p*-value
**Externalizing**					
*Statistical analysis*					
Intent-to-treat	12	0.14	-0.01; 0.29	0.50	0.48
Completer	14	0.21	0.06; 0.37		
*Treatment*					
CBT	15	0.13	-0.01; 0.28	1.07	0.59
CBT plus	5	0.21	-0.05; 0.46		
Emotion-based	5	0.28	0.03; 0.53		
Other	2	-	-		
*Treatment format*					
Group	25	0.17	0.06; 0.27	1.25	0.26
Individual	2	0.46	-0.05; 0.97		
**Internalizing**					
*Statistical analysis*					
Intent-to-treat	6	0.05	-0.14; 0.24	2.57	0.11
Completer	9	0.26	-0.08: 0.45		
*Treatment*					
CBT	7	0.07	-0.09; 0.24	1.82	0.40
Emotion-based	3	0.27	0.03; 0.52		
Other	3	0.08	-0.18; 0.35		
CBT plus	2	-	-		
*Treatment format*					
Group	12	0.16	-0.001; 0.32	0.04	0.85
Individual	3	0.19	-0.15; 0.53		

*k* = number of studies, CI = confidence interval, Qb = test of difference between categories. Statistical analysis = analysis including the whole sample (Intent-to-treat) or those who completed the study (Completer)

### Objective 4: theoretical framework

Small differences in externalizing and internalizing problems were found between programs based on different theoretical frameworks; however, the differences were non-significant (see Tables 6 and 7). When restricting the analysis to programs with at least three studies employing a waitlist control group, the effect sizes for both CBT and Emotion-based treatments increased to approximately 0.30 (see Table 7).

**Table 7:** Effect sizes for CBT- and emotion-based programs vs. waitlist on parent-rated externalizing and internalizing problems

Time point/Outcome	*k*	*d*	95% CI	z-value	Q-value	95% PI	*I*^*2*^ (%)
Post							
CBT							
Externalizing	13	0.30	0.13; 0.46	3.59^c^	32.8^c^	-0.23; 0.83	63.4
Internalizing	6	0.29	0.02; 0.56	2.11^a^	14.25^a^	-0.55; 1.14	64.9
Emotion-based							
Externalizing	4	0.30	0.12; 0.47	3.29^c^	1.73	-	0
Internalizing	2	-	-	-	-	-	-

*k* = number of studies; *d* = Cohen’s *d*; CI = confidence interval; *z* = test of significance for *d*; a = *p* < 0.05; b = *p* < 0.01; c = *p* < 0.001; *Q* = statistical test of heterogeneity; PI = prediction interval; *I*^2^ = proportion of the observed variance that reflects variance in true effects. CBT plus was included in the CBT-based programs. Attachment-based parent training and parent training composed of other theoretical frameworks were too few to evaluate separately. The follow-up studies were too few, when divided into CBT and Emotion-based programs, why there is no follow-up data included in these analyses

In a sensitivity analysis, only minimal changes were seen in *d* for the primary outcomes when different pre/post-correlations (0.1, 0.7, 0.9) were used.

## Discussion

The aim of this meta-analysis was to evaluate the short- and long-term effects of universal parenting programs for parents of 2–17-year-old children in the general population, and to evaluate possible moderators. Small positive short-term effects were obtained for parental ratings of children’s externalizing and internalizing problems, parental stress and parental depression. Subgroup analyses showed that significant effects were only found when interventions were compared to waitlist, not to control groups that may include more active components. Although the long-term between-group effects were positive, they were small and non-significant. Teacher- and child-rated between-group outcomes were not significant. These results will be discussed in turn.

First, the results indicate that the programs have small positive effects on children’s externalizing and internalizing problems. These positive findings are consistent with, though smaller than, the effects typically reported for parental support programs at selected and indicated levels. Such programs often yield small to medium effects on children’s externalizing and internalizing problems, as well as on parental well-being (e.g., [17, 18]). This meta-analysis contributes significantly to the literature by focusing exclusively on universal parenting programs, excluding studies with varying prevention risk levels, and targeting families with children older than 2 years. The mean child age in our sample was approximately 7 years (range 2.6–15 years), whereas most previous meta-analyses centred on families with children aged 0–3 years [76–78] and included samples with other risk levels [76]. Leijten et al. [17], in a meta-analysis of preventive and intervention parenting programs, which included eight CBT-based universal parenting program studies for families with children aged 3–9 years, examined only externalizing problems and found small, non-significant effects for the universal programs. The authors [17] suggested that their non-significant results may be due to floor effects of externalizing problems in that it may be difficult to further reduce already low-level problems. In the present meta-analysis, the significant effect at post assessment on child externalizing problems when comparing all universal programs to a wait-list control group suggests that the measurements were not limited by a floor effect. Importantly, however, there were no significant effects when compared to the active controls, nor any significant follow-up between-group effects. Our within-group effects for child externalizing and internalizing problems were significant and small to medium-sized at post and follow-up assessments, but it should be noted that these within-group comparisons lack control for maturation, natural developmental changes, and non-specific intervention effects. To summarize, the findings of the present meta-analysis are promising in that both externalizing and internalizing child problems were significantly reduced directly after parental participation in a universal parenting program compared to a wait-list control group. The within-group changes suggest that parents participating in parental support groups reported improvements during the intervention period, with continued gains over time. This is important to note, even though families in the control group also appeared to improve, since a significant between-group effect could otherwise reflect deterioration in the control group rather than genuine improvements in the intervention group. Even when the effects of interventions are modest, universal parenting programs may benefit individual children and families by fostering a supportive family environment and preventing the development of mental health problems, thereby reducing risk of future problems on a population level.

We also examined the effect of parental support programs on parental mental health. Research has indicated that children’s externalizing behaviour can have a negative impact on parental stress and depression, and conversely, that parental stress and depression can have a negative impact on children’s externalizing behaviours [15, 24–26]. Our results suggest that parental support programs can reduce this negative interaction, as the programs have small effects on both children’s externalizing behaviour and parents’ stress and depression in the short term. In the meta-analysis by Barlow et al. [23] on parental support programs, also including selective and indicated programs, similar results were obtained, with a small effect on maternal depression and a medium effect on maternal stress, whereas no significant effects were found in their follow-up. In Salari and Enebrink’s [21] systematic review, the results of universal programs on parental mental health were found varied and estimated as unclear. Another meta-analysis, covering the age span from pregnancy to 5 years, evaluated digital, self-delivered universal parenting programs and found small to moderate positive effects on parental depression, anxiety and self-efficacy, but not on stress, satisfaction or parent-child relationship quality [79]. In the present study we only included digital parenting programs if these included therapeutic contact and support. Taken together, the present meta-analysis indicates that at least short-tern effects on parental stress may be better than previously indicated.

The third aim of this meta-analysis was to examine the extent to which the effects on children’s mental health were moderated by factors such as the child’s gender, the parents’ gender, time spent in the program, the number of participants in the study, and the age of the children. Meta-regression was performed on these variables. We found larger effects on both externalizing and internalizing problems with a lower number of study participants. However, after Holm-Bonferroni correction, the moderator was no longer significant for internalizing problems. Previous meta-analyses on parental support programs have similar findings with larger effects for studies with a lower number of participants (e.g., [18]). This effect may be a result of researchers and therapists being more engaged in smaller studies, getting closer to the participants and having improved control of the study. In contrast, larger studies typically require more resources and are therefore often conducted with greater rigor [80].

The subgroup analyses also indicate differences in effects between groups depending on whether studies used a general control group or waiting list as a comparison group. Speculatively, it could be argued that participants on the waiting list not only have an absence of placebo effect, but that they receive a nocebo effect, leading to larger differences between the parental support and waiting list groups. The difference between comparison groups could also be because a minimal intervention in a control group, for example an information brochure, might have a small positive effect. Another possibility is that those who end up in a passive control group or a minimally active control group develop their own strategies to change parts of their lives for the better, to a greater extent than those in a waiting list who know that they soon will be part of an intervention and may act more passively [81].

We also investigated whether the theoretical framework underlying parental support programs influenced their effectiveness. Evaluating the significance of different theoretical frameworks is important for determining which approaches most effectively inform and improve treatment outcomes. Our comparisons did not reveal any significant differences between frameworks. Nevertheless, these findings should be interpreted with caution, given the strong predominance of CBT-based interventions and the relatively small number of studies in the other categories. Consequently, the results should be regarded as preliminary. To our knowledge, no previous comprehensive meta-analysis has examined the relative effectiveness of different theoretical approaches in parental support programs targeting child and parent mental health outcomes. The lack of differences between theoretical frameworks could be due to low power to detect differences as most of the programs included here were based on CBT. For instance, as this meta-analysis shows, most universal parental support programs evaluated in RCT: s are based on CBT, such as Triple P and the Incredible Years. However, the lack of difference could also be because the programs are equally effective. Many programs share overlapping components, which may contribute to comparable outcomes. It might be that reflecting on parenting behaviours in various ways could be enough to promote change in child behaviours [82, 83]. The absence of difference across the theoretical orientations could also be explained by “common factors”, such as therapeutic alliance and practitioner empathy, most often discussed in the psychotherapy literature [84]. These common factors are suggested to contribute to intervention outcomes regardless of theoretical orientation and might be of relevance when programs are delivered in universal populations. However, these suggested explanations require further empirical investigation.

One aspect to consider is that the reduction in problems should be expected to be greater when the problems are greater at baseline [17, 27]. As a result, selective and indicated programs should have larger effect sizes compared to universal programs. Furthermore, universal programs are intended to be given to a general population and not risk or clinical groups, thereby can they reach resourceful families with many protective factors and few risk factors for psychological problems. Among others, Leijten et al. [17] suggest that these programs may have larger long-term preventive effects in reducing risk of future externalizing problems, and as indicated in the current meta-analysis, internalizing problems. This assumption is connected to ideas about the prevention paradox [20], that universal interventions can have a greater effect on counteracting, for example, externalizing problems in the long-term and at the societal level, compared to selective and indicated interventions. However, between-group analyses in the current meta-analysis do not support these assumptions, as no significant effects were observed in the long-term. It should also be noted that these long-term analyses were based on a relatively small number of studies, showed substantial heterogeneity, and are limited to one year in average (ranging from one month to six years). Therefore, further studies with longer follow-up periods are warranted.

### Limitations and strengths

In this meta-analysis, child and parental well-being were defined as primary and secondary outcomes, respectively. We did not include parenting practices as outcomes as these were conceptualized as potential mediators, which may limit the conclusions that can be drawn from the systematic review. The absence of grey literature is also a limitation, as it may result in an incomplete understanding of the available evidence. Several tests of significance were performed, causing an increased risk of a random type 1 error. This was corrected for in the meta-regression. Another limitation is that for some outcomes there were only a small number of studies. For example, at post assessment, we have three studies with child ratings of externalizing problems, three with teacher ratings of externalizing problems and six on parental depression. Further, 15 studies had pre-assessment to follow-up assessments of children’s externalizing problems rated by parents, and only seven on internalizing problems. Many follow-up measurements had substantial dropouts. Thus, the results should be interpreted with caution. As long-term effects are suggested one of the benefits with universal programs, more follow-up studies are needed.

Overall, most studies were of acceptable quality, although several methodological limitations were identified. The risk-of-bias assessment indicated that whereas most studies had some risk of bias, one fifth were rated as high risk. This was partly due to difficulty in blinding outcome assessors, as parent-reported outcomes often are used in parental support programs. Additional concerns include delayed postintervention measurement, not carried out until long after the intervention had ended and insufficiently described randomization process. Although parental ratings represent an established and wide-spread methodology for evaluating outcomes within the parenting-literature, we also acknowledge that this introduces a potential bias as parents both receive the intervention and report on the possible impact of this intervention. Future evaluations of parenting interventions would be improved from establishing other more ecological assessments, e.g. standardized observations of parent-child interactions. To better discern effects over time, we could also have included uncontrolled pre- to post studies and conducted sensitivity analyses comparing these with the RCTs included in the present review.

There are also several strengths in this meta-analysis. We excluded quasi-experimental studies and studies without a comparison group and only included randomized or cluster randomized controlled studies. Included studies also had a relatively high number of participants. Only two studies had fewer than 20 participants per group, most had between 40 and 200 participants in both intervention and comparison groups. We used four different outcomes and separated data to get as detailed results as possible. We excluded studies that used rating scales examining only broad measure of mental health. Data were also not used for studies that pooled estimates of children’s externalizing and internalizing problems, or pooled estimates of parental stress, anxiety, or depression. This allows for a more accurate and unbiased assessment of each outcome. To maintain good quality of structure, collection, and reporting, we used the established standard PRISMA guidelines [28]. Another strength is that we had two different evaluators for each abstract, full text review, and extracted variables, ensuring fewer mistakes.

### Future research

The purpose of universal programs is mainly to be a preventive intervention. Therefore, it is important to examine program effect in the long-term. One third of the included studies lacked follow-up data, and only half of the studies reported follow-up data for both the parenting support group and control group. Thus, there is a shortage of studies on universal programs that examine long-term effects on children’s problem and parents’ mental health. These are required to make an evidence-based decision whether universal programs should be offered on a larger scale in societies. Another approach for future research is to examine which components of the programs are effective, possible mediators and mechanisms, as has been done in several previous meta-analyses on selective or indicated programs (e.g., [17, 85]). In summary, universal parental support programs are a promising, but not yet well researched, field.

## Conclusions

The results indicate that universal parental support programs, at least in the short term and compared to waitlist only, have small positive effects on parent-ratings of children’s externalizing and internalizing problems, as well as on parents’ stress and depression. Thus, this meta-analysis contributes to the empirical basis suggesting that universal parental support programs are valuable for improving children’s and parents’ mental health, with the limitation that the use of parental ratings as outcomes may introduce a potential bias as parents both receive and report on the impact of the intervention. The non-significant findings for long-term between-group effects were based on a relatively small number of studies that showed substantial heterogeneity, underscoring the need for further research. No significant differences emerged between programs with different theoretical frameworks. The literature suggests a link between parental stress and depression and children’s externalizing and internalizing problems. It is therefore particularly encouraging that universal programs appear to have a positive impact on both. In summary, the results for this meta-analysis can be useful for researchers, practitioners and decision-makers who work with parental support.

## Supplementary Information

Supplementary Material 1.

Supplementary Material 2.

## Data Availability

Data supporting the findings of this meta-analysis is available from the corresponding author upon reasonable request.

## Abbreviations

CBT: Cognitive behavioural therapy
PRISMA: Preferred reporting items for systematic reviews and meta-analyses
RCT: Randomized controlled trial
