# Supplementary material for: The effects of universal parental support programs: a systematic review and meta-analysis
Source: BMC Public Health. 2026 Sep 28;26:2941. doi: 10.1186/s12889-026-28844-6 (PMC13656496; doi:10.1186/s12889-026-28844-6)
Supplement: Supplementary file 1 [file 12889_2026_28844_MOESM1_ESM.pdf]

Appendix A:
Documentation of search strategies,
University Library search consultation group

Date: April 2024

Databases:

1. Medline (Ovid)
2. Cochrane Central Register of Controlled Trials (Wiley)
3. Web of Science Core Collection
4. Psycinfo (Ebsco)
5. ERIC (ProQuest)

Total number of hits:

- Before deduplication: 14,467
- After deduplication: 4,714 (dec 2020) + 1,814 (april 2024) = 6,528

<u>Search strategy</u>

A literature search was performed in the following databases: Medline (Ovid), Cochran Central (Wiley), Web of Science Core Collection (Clarivate), Psycinfo (Ebsco) and ERIC (ProQuest).

After the original search was performed on 22 December 2020, the search was last updated on 22 April 2024 using the methods described by Bramer et al (1)

The search strategy was developed in Medline (Ovid) in collaboration with librarians at the Karolinska Institutet University Library. For each search concept Medical Subject Headings (MeSH-terms) and free text terms were identified.

No language restriction was applied and databases were searched from inception.

De-duplication was done using the method described by Bramer et al (2). One final, extra step was added to compare DOIs.

A snow-ball search was applied to check references and citations of eligible studies from the database searches.

The full search strategies for all databases are available in the appendix.

References

1. Bramer W, Bain P. (2017). Updating search strategies for systematic reviews using EndNote. *Journal of the Medical Library Association: JMLA*, 105(3):285-289. doi: 10.5195/jmla.2017.183.

2. Bramer, W. M., Giustini, D., de Jonge, G. B., Holland, L., & Bekhuis, T. (2016). De-duplication of database search results for systematic reviews in EndNote. *Journal of the Medical Library Association: JMLA*, 104(3), 240-243. doi: 10.3163/1536-5050.104.3.014

1. Medline

Interface: Ovid MEDLINE(R) ALL<br><br>Date of Search: 22 April 2024<br><br>Number of hits: 3,457<br><br>Comment: In Ovid, two or more words are automatically searched as phrases; i.e. no quotation marks are needed	Field labels<br><br>• exp/ = exploded MeSH term<br>• / = non exploded MeSH term<br>• .ti,ab,kf. = title, abstract and author keywords<br>• adjx = within x words, regardless of order<br>• * = truncation of word for alternate endings

Database(s): **Ovid MEDLINE(R) ALL** 1946 to April 19, 2024
Search Strategy:

#	Searches	Results
1	exp Parenting/	22001
2	(parent* adj3 (intervention* or program* or train*)).ti,ab,kf.	14989
3	((family* or families) adj2 (train* or intervention* or prevent* or program*)).ti,ab,kf.	21983
4	parenting.ti,ab,kf.	28286
5	(Incredible Years or "Triple P" or "Family Check-Up" or "Tuning into Kids" or "Tuning into Teens" or "123 Magic Years" or "Family Links Nurturing Programme").ti,ab,kf.	660
6	or/1-5	66153
7	exp Child Behavior/	27566
8	exp Adolescent Behavior/	35590
9	Child Behavior Disorders/	20894
10	Conduct Disorder/	3970
11	Child Health/	5489
12	Adolescent Health/	2011
13	Anxiety/	112205
14	Depression/	156940
15	Internal-External Control/	18017
16	exp Self-Injurious Behavior/	86113
17	((behavior* or behaviour*) adj2 (aggressi* or antisocial* or conduct* or difficult* or externali* or internali* or oppositional* or problem*)).ti,ab,kf.	66570
18	((adolescen* or child* or infant* or teen*) adj2 (aggressi* or antisocial* or behavio* or emotion* or externali* or health* or internali* or misconduct* or oppositional* or problem* or psychopathol* or well-being)).ti,ab,kf.	201379
19	((conduct or externali* or internali*) adj2 (difficult* or problem* or symptom*)).ti,ab,kf.	15513

20	((behaviour* or behavior* or conduct* or oppositional*) adj2 disorder*).ti,ab,kf.	28942
21	(anxiety or anxious* or depress* or self-cut* or self-destructive* or self-harm* or self-injur* or suicid*).ti,ab,kf.	810953
22	or/7-21	1162144
23	exp Child/	2199897
24	Infant/	878811
25	Adolescent/	2241147
26	(adolescen* or babies or baby or boy or child* or girl? or infan* or juvenil* or kid? or paediatric* or peadiatric* or pediatric* or teen* or toddler? or youth*).ti,ab,kf.	2746193
27	or/23-26	4608890
28	Program Development/	30364
29	Program Evaluation/	67573
30	((empower* or decreas* or improv* or increas* or promot* or prevent* or reduc*) and (educat* or intervention* or program* or train*)).ti,ab,kf.	2049697
31	(program* adj2 (develop* or effect* or evaluat*)).ti,ab,kf.	79063
32	or/28-31	2115310
33	randomized controlled trial.pt.	611126
34	controlled clinical trial.pt.	95522
35	(randomised or randomized or randomly).ab.	1123250
36	placebo.ab.	247523
37	clinical trials as topic.sh.	202068
38	trial.ti.	307264
39	or/33-38	1642094
40	6 and 22 and 27 and 32 and 39	3475
41	remove duplicates from 40	3457

2. Cochrane Central Register of Controlled Trials

Interface: Wiley<br><br>Date of Search: 22 April 2024<br><br>Number of hits: 4,052	Field labels<br><br>• ti,ab,kw = title, abstract and author keywords<br>• NEAR/x = within x words, regardless of order<br>• * = truncation of word for alternate endings

ID	Search	Hits
#1	(parent* NEAR/3 (intervention* or program* or train*)):ab,ti,kw	8031
#2	((family* or "families") NEAR/2 (train* or intervention* or prevent* or program*)):ab,ti,kw	5326
#3	parenting:ab,ti,kw	5903
#4	(“Incredible Years” or "Triple P" or "Family Check-Up" or "Tuning into Kids" or "Tuning into Teens" or "123 Magic Years" or "Family Links Nurturing Programme"):ab,ti,kw	610
#5	#1 or #2 or #3 or #4	14931
#6	((behavior* or behaviour*) NEAR/2 (aggressi* or antisocial* or conduct* or difficult* or externali* or internali* or oppositional* or problem*)):ab,ti,kw	6981
#7	((adolescen* or child* or infant* or teen*) NEAR/2 (aggressi* or antisocial* or behavio* or emotion* or externali* or health* or internali* or misconduct* or oppositional* or problem* or psychopathol* or "well-being")):ab,ti,kw	32113
#8	((conduct or externali* or internali*) NEAR/2 (difficult* or problem* or symptom*)):ab,ti,kw	1730
#9	((behaviour* or behavior* or conduct* or oppositional*) NEAR/2 disorder*):ab,ti,kw	8390
#10	(anxiety or anxious* or depress* or self-cut* or self-destructive* or self-harm* or self-injur* or suicid*):ab,ti,kw	155079
#11	#6 or #7 or #8 or #9 or #10	187728
#12	(adolescen* or babies or baby or boy? or child* or girl? or infan* or juvenil* or kid? or paediatric* or peadiatric* or pediatric* or teen* or toddler? or youth*):ab,ti,kw	365079
#13	((empower* or decreas* or improv* or increas* or promot* or prevent* or reduc*) and (educat* or intervention* or program* or train*)):ab,ti,kw	472787
#14	(program* NEAR/2 (develop* or effect* or evaluat*)):ab,ti,kw	21264
#15	#13 or #14	476044

#16	#5 AND #11 AND #12 AND #15	5703
#17	(clinicaltrials or trialsearch):so	499880
#18	#16 NOT #17 4052	

3. Web of Science Core Collection

Interface: Clarivate Analytics<br><br>Date of Search: 22 April 2024<br><br>Number of hits: 3,012	Field labels<br><br>• TI = title<br>• AB = abstract<br>• AK = author keywords<br>• NEAR/x = within x words, regardless of order<br>• * = truncation of word for alternate endings<br><br>Note: sometimes "quotation marks" are needed for single search terms to avoid automatic term mapping (lemmatization).

#	Search Query	Results
1	TI=((parent* NEAR/2 (intervention* or program* or train*))) OR AB=((parent* NEAR/2 (intervention* or program* or train*))) OR AK=((parent* NEAR/2 (intervention* or program* or train*)))<br>OR TI=(((family* or families) NEAR/1 (train* or intervention* or prevent* or program*))) OR AB=(((family* or families) NEAR/1 (train* or intervention* or prevent* or program*))) OR AK=(((family* or families) NEAR/1 (train* or intervention* or prevent* or program*)))<br>OR TI=("Parenting") OR AB=("Parenting") OR AK=("Parenting")<br>OR TI=((“Incredible Years” or "Triple P" or "Family Check-Up" or "Tuning into Kids" or "Tuning into Teens" or "123 Magic Years" or "Family Links Nurturing Programme")) OR AB=((“Incredible Years” or "Triple P" or "Family Check-Up" or "Tuning into Kids" or "Tuning into Teens" or "123 Magic Years" or "Family Links Nurturing Programme")) OR AK=((“Incredible Years” or "Triple P" or "Family Check-Up" or "Tuning into Kids" or "Tuning into Teens" or "123 Magic Years" or "Family Links Nurturing Programme"))	74564
2	TI=(((behavior* or behaviour*) NEAR/1 (aggressi* or antisocial* or conduct* or difficult* or externali* or internali* or oppositional* or problem*))) OR AB=(((behavior* or behaviour*) NEAR/1 (aggressi* or antisocial* or conduct* or difficult* or externali* or internali* or oppositional* or problem*))) OR AK=(((behavior* or behaviour*) NEAR/1 (aggressi* or antisocial* or conduct* or difficult* or externali* or internali* or oppositional* or problem*)))<br>OR TI=(((adolescen* or child* or infant* or teen*) NEAR/1 (aggressi* or antisocial* or behavio* or emotion* or externali* or health* or internali* or misconduct* or oppositional* or problem* or psychopathol* or “well-being”)))<br>OR AB=(((adolescen* or child* or infant* or teen*) NEAR/1 (aggressi* or antisocial* or behavio* or emotion* or externali* or health* or internali* or misconduct* or oppositional* or problem* or psychopathol* or “well-being”)))<br>OR AK=(((adolescen* or child* or infant* or teen*) NEAR/1 (aggressi* or antisocial* or behavio* or emotion* or externali* or health* or internali* or misconduct* or oppositional* or problem* or psychopathol* or “well-being”)))<br>OR TI=(((“conduct” or externali* or internali*) NEAR/1 (difficult* or problem* or symptom*))) OR AB=(((“conduct” or externali* or internali*) NEAR/1 (difficult* or problem* or symptom*))) OR AK=(((“conduct” or externali* or internali*) NEAR/1 (difficult* or problem* or symptom*)))	1188902

	OR TI=(((behaviour* or behavior* or conduct* or oppositional*) NEAR/1 disorder*)) OR AB=(((behaviour* or behavior* or conduct* or oppositional*) NEAR/1 disorder*)) OR AK=(((behaviour* or behavior* or conduct* or oppositional*) NEAR/1 disorder*)) OR TI=((“anxiety” or anxious* or depress* or self-cut* or self-destructive* or self-harm* or self-injur* or suicid*)) OR AB=((“anxiety” or anxious* or depress* or self-cut* or self-destructive* or self-harm* or self-injur* or suicid*)) OR AK=((“anxiety” or anxious* or depress* or self-cut* or self-destructive* or self-harm* or self-injur* or suicid*))	
3	TI=((adolescen* or "babies" or "baby" or boy$ or child* or girl$ or infan* or juvenil* or kid$ or paediatric* or peadiatric* or pediatric* or teen* or toddler$ or youth*)) OR AB=((adolescen* or "babies" or "baby" or boy$ or child* or girl$ or infan* or juvenil* or kid$ or paediatric* or peadiatric* or pediatric* or teen* or toddler$ or youth*)) OR AK=((adolescen* or "babies" or "baby" or boy$ or child* or girl$ or infan* or juvenil* or kid$ or paediatric* or peadiatric* or pediatric* or teen* or toddler$ or youth*))	3129378
4	TI=(((empower* or decreas* or improv* or increas* or promot* or prevent* or reduc*) and (educat* or intervention* or program* or train*))) OR AB=(((empower* or decreas* or improv* or increas* or promot* or prevent* or reduc*) and (educat* or intervention* or program* or train*))) OR AK=(((empower* or decreas* or improv* or increas* or promot* or prevent* or reduc*) and (educat* or intervention* or program* or train*)))	2470606
5	TI=(("randomised" or "randomized" or "randomly" or "placebo")) OR AB=(("randomised" or "randomized" or "randomly" or "placebo")) OR TI=("trial")	1530881
6	#5 AND #4 AND #3 AND #2 AND #1	3012

4. Psycinfo

Interface: Ebsco<br><br>Date of Search: 22 April 2024<br><br>Number of hits: 3,219	Field labels<br><br>• DE = subject heading<br>• TI = title<br>• AB = abstract<br>• KW = author keywords<br>• Nx = within x words, regardless of order<br>• * = truncation of word for alternate endings

#	Query	Results
S38	S36 OR S37	3,219
S37	S7 AND S25 AND S30 AND S35<br>Limiters - Age Groups: Childhood (birth-12 yrs), Adolescence (13-17 yrs)	2,385
S36	S7 AND S25 AND S26 AND S30 AND S35	3,195
S35	S31 OR S32 OR S33 OR S34	195,648
S34	TI trial	45,634
S33	DE "Randomized Controlled Trials" OR DE "Randomized Clinical Trials" OR DE "Clinical Trials" OR DE "Randomized Controlled Trials"	13,610
S32	TI placebo OR AB placebo	36,937
S31	TI ((randomised or randomized or randomly)) OR AB ((randomised or randomized or randomly))	164,378
S30	S27 OR S28 OR S29	660,450
S29	TI (((empower* or decreas* or improv* or increas* or promot* or prevent* or reduc*) and (educat* or intervention* or program* or train*))) OR AB (((empower* or decreas* or improv* or increas* or promot* or prevent* or reduc*) and (educat* or intervention* or program* or train*))) OR KW (((empower* or decreas* or improv* or increas* or promot* or prevent* or reduc*) and (educat* or intervention* or program* or train*)))	644,635
S28	DE "Program Evaluation" OR DE "Educational Program Evaluation" OR DE "Mental Health Program Evaluation"	29,860
S27	DE "Program Development"	11,900
S26	TI ((adolescen* OR babies OR baby OR boy# OR child* OR girl# OR infan* OR juvenil* OR kid# OR paediatric* OR peadiatric* OR pediatric* OR teen* OR toddler# OR youth*)) OR AB ((adolescen* OR babies OR baby OR boy# OR child* OR girl# OR infan* OR juvenil* OR kid# OR paediatric* OR peadiatric* OR pediatric* OR teen* OR toddler# OR youth*)) OR KW ((adolescen* OR babies OR baby OR boy# OR child* OR girl# OR infan* OR juvenil* OR kid# OR paediatric* OR peadiatric* OR pediatric* OR teen* OR toddler# OR youth*))	930,319
S25	S8 OR S9 OR S10 OR S11 OR S12 OR S13 OR S14 OR S15 OR S16 OR S17 OR S18 OR S19 OR S20 OR S21 OR S22 OR S23 OR S24	704,646
S24	TI ((anxiety or anxious* or depress* or "self-cut*" or "self-destructive*" or "self-harm*" or "self-injur*" or suicid*)) OR AB ((anxiety or anxious* or depress* or "self-cut*" or "self-destructive*" or "self-harm*" or "self-injur*" or suicid*)) OR KW ((anxiety or anxious* or depress* or "self-cut*" or "self-destructive*" or "self-harm*" or "self-injur*" or suicid*))	477,581
S23	TI (((behaviour* or behavior* or conduct* or oppositional*) N2 disorder*)) OR AB (((behaviour* or behavior* or conduct* or oppositional*) N2 disorder*)) OR KW (((behaviour* or behavior* or conduct* or oppositional*) N2 disorder*))	31,280
S22	TI (((conduct or externali* or internali*) N2 (difficult* or problem* or symptom*))) OR AB (((conduct or externali* or internali*) N2 (difficult* or problem* or symptom*))) OR KW (((conduct or externali* or internali*) N2 (difficult* or problem* or symptom*)))	20,286
S21	TI (((adolescen* or child* or infant* or teen*) N2 (aggressi* or antisocial* or behavio* or emotion* or externali* or health* or internali* or misconduct* or	163,139

	oppositional* or problem* or psychopathol* or "well-being"))) OR AB (((adolescen* or child* or infant* or teen*) N2 (aggressi* or antisocial* or behavio* or emotion* or externali* or health* or internali* or misconduct* or oppositional* or problem* or psychopathol* or "well-being"))) OR KW (((adolescen* or child* or infant* or teen*) N2 (aggressi* or antisocial* or behavio* or emotion* or externali* or health* or internali* or misconduct* or oppositional* or problem* or psychopathol* or "well-being")))	
S20	TI (((behavior* or behaviour*) N2 (aggressi* or antisocial* or conduct* or difficult* or externali* or internali* or oppositional* or problem*))) OR AB (((behavior* or behaviour*) N2 (aggressi* or antisocial* or conduct* or difficult* or externali* or internali* or oppositional* or problem*))) OR KW (((behavior* or behaviour*) N2 (aggressi* or antisocial* or conduct* or difficult* or externali* or internali* or oppositional* or problem*)))	84,708
S19	DE "Depression (Emotion)"	17,214
S18	DE "Anxiety"	85,601
S17	DE "Adolescent Health"	4,098
S16	DE "Child Health"	1,630
S15	DE "Internalizing Symptoms"	1,520
S14	DE "Internalization" OR DE "Introjection"	5,912
S13	DE "Externalizing Symptoms"	754
S12	DE "Externalization"	5,452
S11	DE "Behavior Problems" OR DE "Tantrums"	27,587
S10	(((((((DE "Behavior Disorders" OR DE "Conduct Disorder" OR DE "Disruptive Behavior Disorders" OR DE "Emotional and Behavioral Disorders" OR DE "Impulse Control Disorders" OR DE "Kleptomania" OR DE "Oppositional Defiant Disorder" OR DE "Pyromania" OR DE "Self-Destructive Behavior") OR (DE "Disruptive Behavior Disorders" OR DE "Conduct Disorder" OR DE "Oppositional Defiant Disorder")) OR (DE "Emotional and Behavioral Disorders" OR DE "Emotional Disturbances")) OR (DE "Impulse Control Disorders" OR DE "Explosive Disorder")) OR (DE "Self-Destructive Behavior" OR DE "Nonsuicidal Self-Injury" OR DE "Suicidal Behavior")) OR (DE "Nonsuicidal Self-Injury" OR DE "Head Banging" OR DE "Self-Inflicted Wounds" OR DE "Self-Poisoning")) OR (DE "Suicidal Behavior" OR DE "Attempted Suicide" OR DE "Suicidal Ideation" OR DE "Suicide")) OR (DE "Suicide" OR DE "Military Suicide" OR DE "Youth Suicide")	72,512
S9	DE "Adolescent Behavior"	19,305
S8	DE "Child Behavior"	8,039
S7	S1 OR S2 OR S3 OR S4 OR S5 OR S6	147,634
S6	TI (("Incredible Years" or "Triple P" or "Family Check-Up" or "Tuning into Kids" or "Tuning into Teens" or "123 Magic Years" or "Family Links Nurturing Programme")) OR AB (("Incredible Years" or "Triple P" or "Family Check-Up" or "Tuning into Kids" or "Tuning into Teens" or "123 Magic Years" or "Family Links Nurturing Programme")) OR KW (("Incredible Years" or "Triple P" or "Family Check-Up" or "Tuning into Kids" or "Tuning into Teens" or "123 Magic Years" or "Family Links Nurturing Programme")) OR TM (("Incredible Years" or "Triple P" or "Family Check-Up" or "Tuning into Kids" or "Tuning into Teens" or "123 Magic Years" or "Family Links Nurturing Programme"))	973
S5	TI parenting OR AB parenting OR KW parenting OR TM parenting	48,311
S4	TI (((family* or families) N2 (train* or intervention* or prevent* or program*))) OR AB (((family* or families) N2 (train* or intervention* or prevent* or program*))) OR KW (((family* or families) N2 (train* or intervention* or prevent* or program*))) OR TM (((family* or families) N2 (train* or intervention* or prevent* or program*)))	19,835
S3	TI ((parent* N3 (intervention* or program* or train*))) OR AB ((parent* N3 (intervention* or program* or train*))) OR KW ((parent* N3 (intervention* or program* or train*))) OR TM((parent* N3 (intervention* or program* or train*))))	22,013
S2	DE "Parent Training"	6,590

S1	DE "Parenting" OR DE "Childrearing Practices" OR DE "Coparenting" OR DE "Parent Child Communication" OR DE "Parent Child Relations" OR DE "Parental Involvement" OR DE "Parenthood Status" OR DE "Parenting Skills" OR DE "Parenting Style" OR DE "Childrearing Practices" OR DE "Child Care" OR DE "Child Discipline" OR DE "Physical Discipline" OR DE "Toilet Training" OR DE "Weaning" OR DE "Child Care" OR DE "Child Care Workers" OR DE "Child Day Care" OR DE "Child Self Care" OR DE "Parental Attitudes" OR DE "Parental Expectations" OR DE "Parent Child Relations" OR DE "Father Child Relations" OR DE "Mother Child Relations" OR DE "Parental Attitudes" OR DE "Parental Involvement" OR DE "Parent School Relationship" OR DE "Parenthood Status" OR DE "Childlessness" OR DE "Parenting Style" OR DE "Authoritarian Parenting" OR DE "Authoritative Parenting" OR DE "Permissive Parenting"	106,928

5. ERIC

Interface: ProQuest<br><br>Date of Search: 22 April 2024<br><br>Number of hits: 727	Field labels<br><br>• MAINSUBJECT.EXACT.EXPLODE = exploded subject heading<br>• MAINSUBJECT.EXACT non exploded subject heading<br>• TI,AB = title, abstract<br>• N/x = within x words, regardless of order<br>• * = truncation of word for alternate endings

#1 MAINSUBJECT.EXACT("Parent Education") OR TI,AB(parent* NEAR/3 (intervention* OR program* OR train*)) OR TI,AB((family* OR families) NEAR/2 (train* OR intervention* OR prevent* OR program*)) OR TI,AB("Parenting") OR TI,AB("Incredible Years" OR "Triple P" OR "Family Check-Up" OR "Tuning into Kids" OR "Tuning into Teens" OR "123 Magic Years" OR "Family Links Nurturing Programme")

#2 MAINSUBJECT.EXACT.EXPLODE("Child Behavior") OR MAINSUBJECT.EXACT("Behavior Problems" OR "Self Destructive Behavior" OR "Suicide" OR "Child Health" OR "Anxiety" OR "Depression (Psychology)" OR "Emotional Disturbances") OR TI,AB(aggressi* OR antisocial* OR behavio* OR emotion* OR conduct* OR difficult* OR externali* OR internali* OR misconduct* OR oppositional* OR problem* OR psychopathol* OR "well-being" OR anxiety OR anxious* OR depress* OR self-cut* OR self-destructive* OR self-harm* OR self-injur* OR suicid*)

#3 MAINSUBJECT.EXACT("Children" OR "Adolescents") OR TI,AB(adolescen* OR babies OR baby OR boy? OR child* OR girl? OR infan* OR juvenil* OR kid? OR paediatric* OR peadiatric* OR pediatric* OR teen* OR toddler? OR youth*)

#4 MAINSUBJECT.EXACT("Program Development" OR "Program Implementation" OR "Program Evaluation" OR "Program Effectiveness") OR TI,AB((empower* OR decreas* OR improv* OR increas* OR promot* OR prevent* OR reduc*) AND (educat* OR intervention* OR program* OR train*)) OR TI,AB(program* NEAR/2 (develop* OR effect* OR evaluat*))

#5 MAINSUBJECT.EXACT("Randomized Controlled Trials") OR TI,AB(randomised OR randomized OR randomly OR placebo) OR TI(trial)

#1 AND #2 AND #3 AND #4 AND #5

Appendix B: Formulation of inclusion- and exclusion criteria

Inclusion criteria

To be included in the meta-analysis the study must:

1. Be published in a scientific journal and written in English
2. Include parents (or other primary caregiver) from a general population, with children in the age of 2–17 years. The parents should not be in a risk group, and the children should be in a risk group or have a clinical diagnosis.
3. Used parental support program should have a psychological theorical basis and the purpose of influencing parental strategies.
4. Have therapeutic contact, either face-to-face, via internet/portal or by telephone
5. Be a randomized controlled trial or cluster randomized controlled trial. No quasi-controlled studies.
6. Have an outcome measure with a continuous scale of externalizing and/or internalizing problems in children. This included self-harm and suicidal behaviours but not measures of abuse, eating disorders or obesity.
7. Have intervention- and comparison groups with at least 10 participants in each group.

Exclusion criteria

Studies are excluded from this meta-analysis if:

1. The study is a secondary analysis of a previously published study (follow-up studies are not excluded).
2. The intervention is only based on self-help literature or have a pre-recorded video-based setup.
3. Data (*M* and *SD/SE* or *d*) is not available.

Appendix C: Excluded studies during full text review

Table 8

Number of studies that were excluded due to not meeting inclusion criteria

Inclusion- and exclusion criteria	*n* (totally 414)
Universal sample	184
Parental support training (only)	92
Age of children: 2–17 years	31
Measurement of children's externalizing/internalizing problems	26
Not a doctoral dissertation	18
Original study (not secondary analysis)	17
Published in English	12
RCT[a]	11
Therapeutic contact (not only self-help)	10
≥10 participants in intervention and comparison group	7
Primary caregiver[b]	3
Data: *M* and *SD/SE* or *d* is reported[c]	1
Wrong publication, not a chapter in book	1
Study not retracted	1

Note. Most of these studies did not reach several of the criteria, but only the first noted reason for exclusion is presented; a = RCT = randomized controlled study; b = the programs should include the primary caregivers in the intervention; c = *M* = mean, *SD* = standard deviation, *SE* = standard error and *d* = standardized difference of mean.

Appendix D

Risk of bias assessment for each criterion

Author(s)	Year	Item 1	Item 2	Item 3	Item 4	Item 5	Total
Altafim	2019	Low	High	Some	High	Some	High
Amador Buenabad	2020	Low (cluster)	Low	Low	Some	Some	Some
Bodenmann	2008	Some	Low	Low	High	Some	High
Burke	2012	Low	Low	Low	High	Some	High
Burkhardt	2024	Low	Some	Low	Some	Some	Some
Chu	2015	Low	Low	Low	Some	Some	Some
Dadds	2008	Low (cluster)	Low	Some	Some	Some	Some
Foskolos	2023	High	Some	High	High	Low	High
Grolnick	2021	Low	Low	Low	Some	Some	Some
Hahlweg	2008	Low	Low	Some	Some	Some	Some
Hahlweg	2010	Low (cluster)	Low	Low	Some	Some	Some
Havighurst	2010	Low (cluster)	Some	Some	Some	Some	Some
Kehoe	2014	Low (cluster)	Low	Low	Some	Some	Some
Leung	2017	Low (cluster)	Low	Some	Low	Some	Some
Leung	2023	Some	Low	Some	Some	Low	Some
Lösel	2009	Low (cluster)	Some	Low	Some	Some	Some
MacKenzie	2000	Low	Low	Some	Some	Some	Some
Malti	2011	Low (cluster)	Low	Some	Some	Some	Some
Matsumoto	2007	Low	Some	Low	Some	Some	Some
Nitsch	2015	Low	Low	Some	Some	Some	Some
Ozturk	2019	Low	Low	Low	Some	Some	Some
Qui	2022	Low	Low	Some	Some	Some	Some
Reedtz	2011	Low	Low	Low	Some	Some	Some
Rincon	2018	Low (cluster)	Low	Low	Low	Some	Some
Sampaio	2015	High (cluster)	Low	Low	Some	Some	High
Schilling	2020	Low	Low	Low	Some	Low	Some
Smith	2018	Low	Low	High	High	Low	High
Sumargi	2014	Low	Low	Low	Some	Low	Some
Webster-Stratton	1982	Low	Low	Low	Some	Some	Some
Wilson	2012	Low (cluster)	Low	Low	Some	Some	Some
Yap	2018	Low	Low	Low	Low	Some	Some

Note: Assessment by the Cochrane RoB 2 tool for randomized trials, the original version for individually randomized trials and a custom version for the cluster randomized trials; cluster = cluster randomization.
