# Supplementary material for: The effects of universal parental support programs: a systematic review and meta-analysis
Source: BMC Public Health. 2026 Sep 28;26:2941. doi: 10.1186/s12889-026-28844-6 (PMC13656496; doi:10.1186/s12889-026-28844-6)
Supplement: Supplementary file 2 [file 12889_2026_28844_MOESM2_ESM.pdf]

Supplementary Figures: Forest plots of between-group differences between universal parent support and a control group.

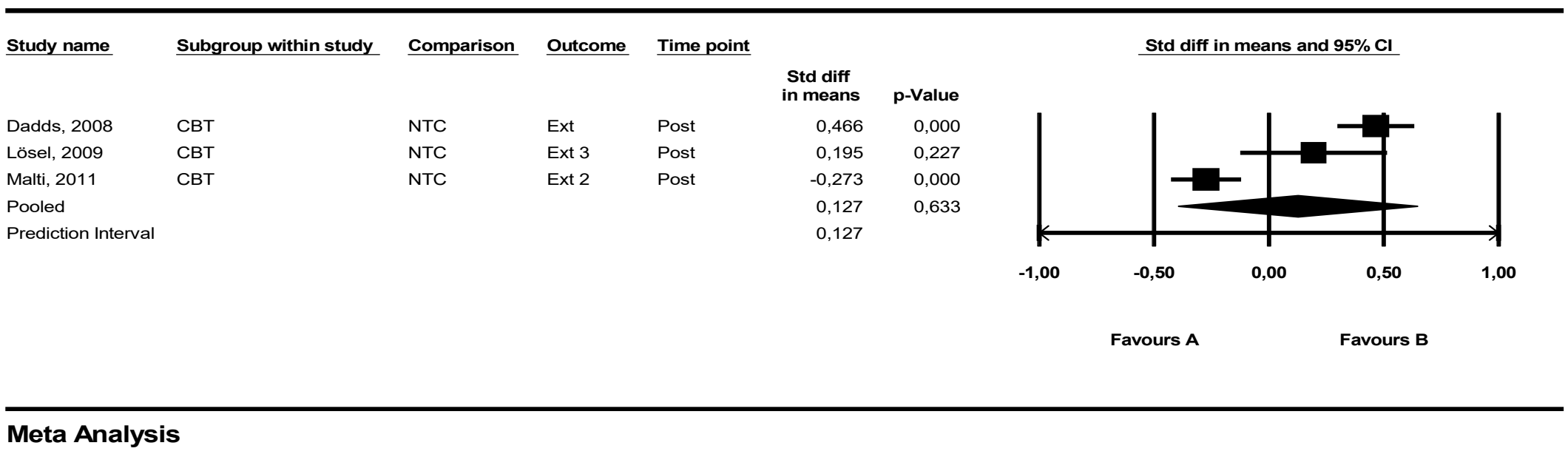

Figure S1. Forest plot of standardized mean differences in teacher ratings of externalizing behaviours at post assessment across included studies. NTC = no treatment control; Ext = externalizing problems.

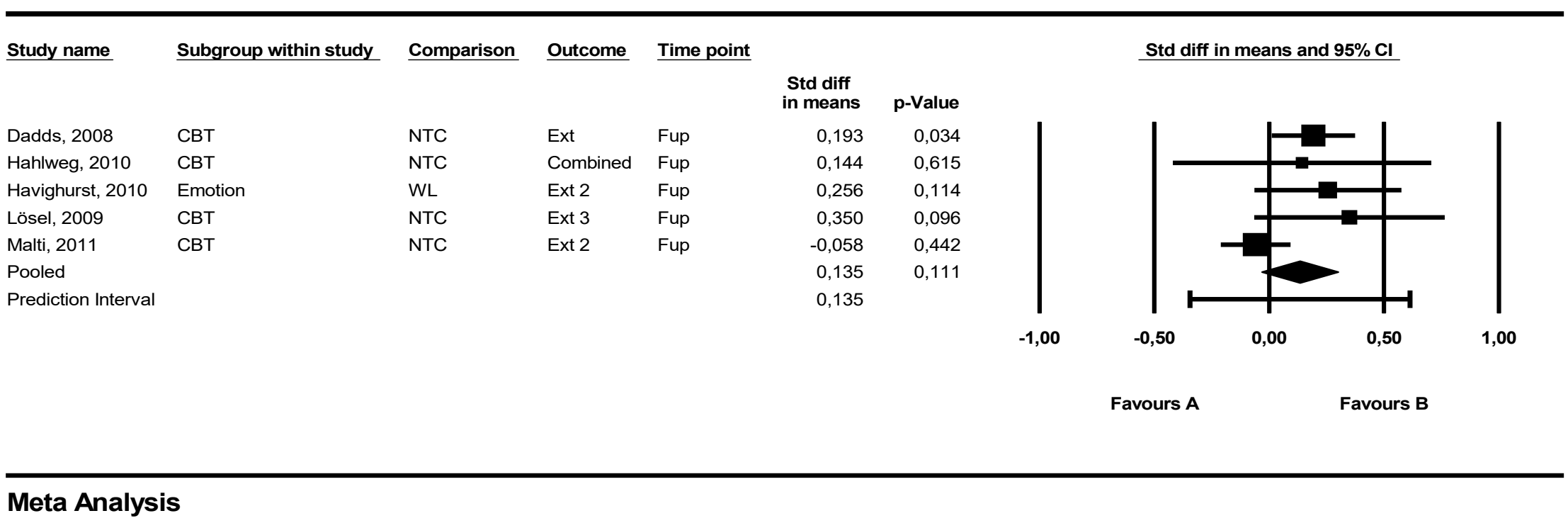

Figure S2. Forest plot of standardized mean differences in teacher ratings of externalizing behaviours at follow-up across included studies. NTC = no treatment control; WL = waitlist control; Ext = externalizing problems; Fup = follow up measurement.

Study name	Subgroup within study	Comparison	Outcome	Time point	Std diff in means	p-Value
Chu, 2015	CBT	WL	Ext	Post	0,139	0,557
Kehoe, 2014	Emotion	NTC	Ext 2	Post	0,166	0,227
Malti, 2011	CBT	NTC	Ext 3	Post	0,000	1,000
Pooled					0,046	0,471
Prediction Interval						

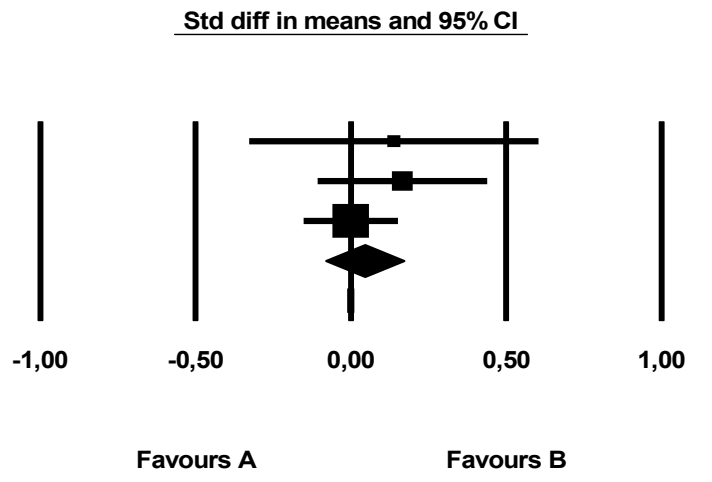

Meta Analysis

Figure S3. Forest plot of standardized mean differences in child ratings of externalizing behaviour at post assessment across included studies. WL = waitlist control; NTC = no treatment control; Ext = externalizing problems.

Study name	Subgroup within study	Comparison	Outcome	Time point	Std diff in means	p-Value
Chu, 2015	CBT	WL	Ext	Fup	0,647	0,008
Lösel, 2009 (2013)	CBT	NTC	Ext 2	Fup	0,490	0,080
Malti, 2011 (Averdijk, 2016)	CBT	NTC	Ext 4	Fup	-0,059	0,480
Pooled					0,316	0,224
Prediction Interval					0,316	

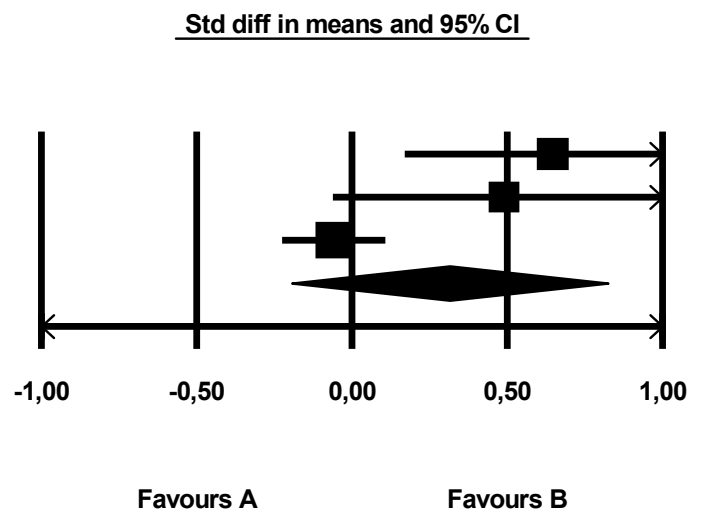

Meta Analysis

Figure S4. Forest plot of standardized mean differences in child ratings of externalizing behaviour at follow-up across included studies. WL = waitlist control; NTC = no treatment control; Ext = externalizing problems; Fup = follow up measurement.

Study name	Subgroup within study	Comparison	Outcome	Time point	Std diff in means	p-Value
Bodenmann, 2008	CBT	NTC	Combined	Fup	0,234	0,244
Burkhardt, 2024	Emotion	WL	Ext	Fup	0,274	0,128
Foskolos, 2023	CBT	WL	Combined	Fup	0,320	0,070
Hahlweg, 2010	CBT	NTC	Combined	Fup	-0,126	0,521
Havighurst, 2010	Emotion	WL	Ext 1	Fup	0,340	0,027
Lösel, 2009	CBT	NTC	Ext 1	Fup	-0,166	0,342
MacKenzie, 2000	CBT	NTC	Ext	Fup	0,507	0,165
Malti, 2011	CBT	NTC	Ext 1	Fup	0,027	0,719
Reedtz, 2011	CBT	TAU	Ext	Fup	-0,189	0,318
Sampaio, 2015	CBT	WL	Ext	Fup	-0,115	0,308
Smith, 2018	CBT	Pla/AC	Ext	Fup	0,647	0,000
Pooled					0,130	0,101
Prediction Interval					0,130	

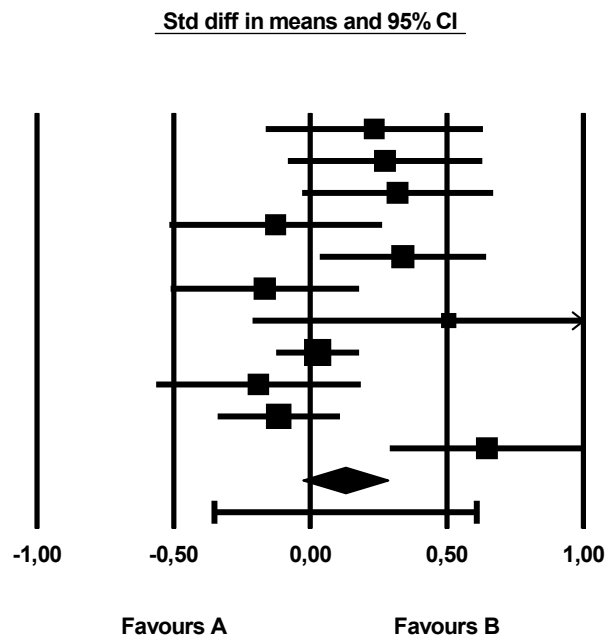

Meta Analysis

Figure S5. Forest plot of standardized mean differences in parent ratings of externalizing behaviours at follow-up across included studies. WL = waitlist control; NTC = no treatment control; TAU = treatment as usual; Pla/AC = placebo-attention control; Ext = externalizing problems; Fup = follow up measurement.

Study name	Subgroup within study	Comparison	Outcome	Time point	Std diff in means	p-Value
Burkhardt, 2024	Emotion	WL	Int	Fup	-0,035	0,845
Foskolos, 2023	CBT	WL	Int	Fup	0,210	0,189
Hahlweg, 2010	CBT	NTC	Combined	Fup	-0,179	0,366
Lösel, 2009	CBT	NTC	Int 1	Fup	-0,236	0,176
Smith, 2018	CBT	Pla/AC	Int	Fup	0,510	0,011
Yap, 2018	Other	Pla/AC	Combined	Fup	0,144	0,173
Pooled					0,072	0,469
Prediction Interval					0,072	

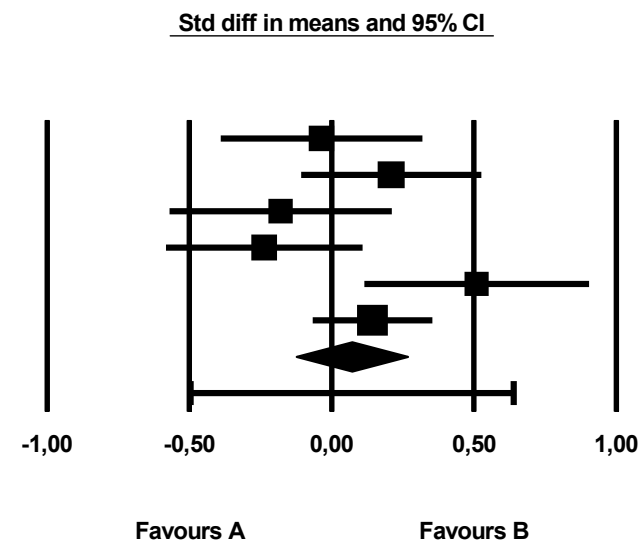

Meta Analysis

Figure S6. Forest plot of standardized mean differences in parent ratings of internalizing behaviour problems at follow-up across included studies. WL = waitlist control; NTC = no treatment control; Pla/AC = placebo-attention control; Ext = internalizing problems; Fup = follow up measurement.

Study name	Subgroup within study	Comparison	Outcome	Time point	Std diff in means	p-Value
Burke, 2012	CBT	WL	Par-S	Post	0,202	0,225
Chu, 2015	CBT	WL	Par-S	Post	0,157	0,507
Leung, 2017	CBT	WL	Par-S	Post	0,479	0,003
Leung, 2023	CBT	WL	Par-S	Post	0,086	0,514
MacKenzie, 2000	CBT	NTC	Par-S	Post	0,287	0,426
Matsumoto, 2007	CBT	WL	Par-S	Post	0,301	0,290
Nitsch, 2015	CBT	WL	Par-S	Post	0,770	0,000
Ozturk, 2019	Attachment	WL	Par-S	Post	-0,410	0,180
Sampaio, 2015	CBT	WL	Par-S	Post	0,072	0,348
Pooled					0,221	0,015
Prediction Interval					0,221	

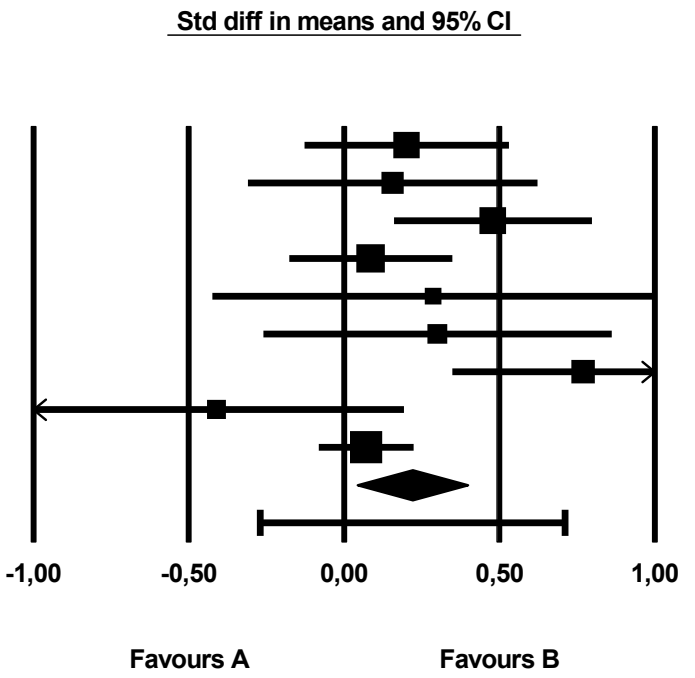

Meta Analysis

Figure S7. Forest plot of standardized mean differences in parent ratings of parental stress at post assessment across included studies. WL = waitlist control; NTC = no treatment control; Par-S = parental stress.

Study name	Subgroup within study	Comparison	Outcome	Time point	Std diff in means	p-Value
Chu, 2015	CBT	WL	Par-S	Fup	-0,087	0,711
MacKenzie, 2000	CBT	NTC	Par-S	Fup	0,414	0,254
Sampaio, 2015	CBT	WL	Par-S	Fup	0,024	0,757
Pooled					0,028	0,689
Prediction Interval						

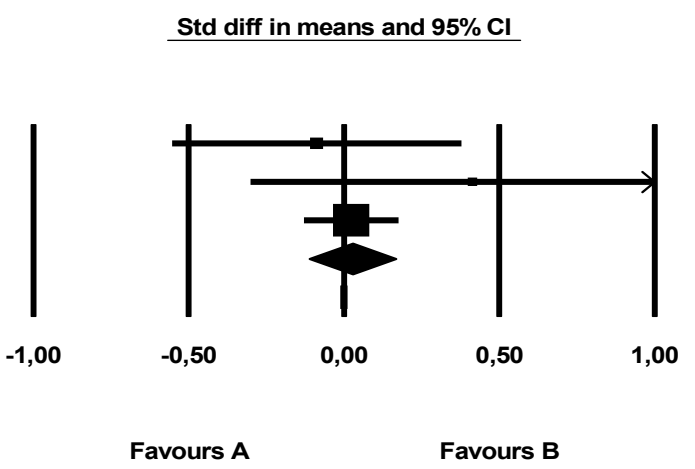

Meta Analysis

Figure S8. Forest plot of standardized mean differences in parent ratings of parental stress at follow-up across included studies. WL = waitlist control; NTC = no treatment control; Par-S = parental stress; Fup = follow up measurement.

Study name	Subgroup within study	Comparison	Outcome	Time point	Std diff in means	p-Value
Burke, 2012	CBT	WL	Par-D	Post	0,213	0,201
Chu, 2015	CBT	WL	Par-D	Post	0,599	0,013
Hahlweg, 2008	CBT	WL	Combined	Post	0,332	0,274
Matsumoto, 2007	CBT	WL	Par-D	Post	0,119	0,673
Rincon, 2018	CBT	WL	Par-D	Post	0,074	0,501
Sampaio, 2015	CBT	WL	Par-D	Post	0,063	0,414
Pooled					0,132	0,032
Prediction Interval					0,132	

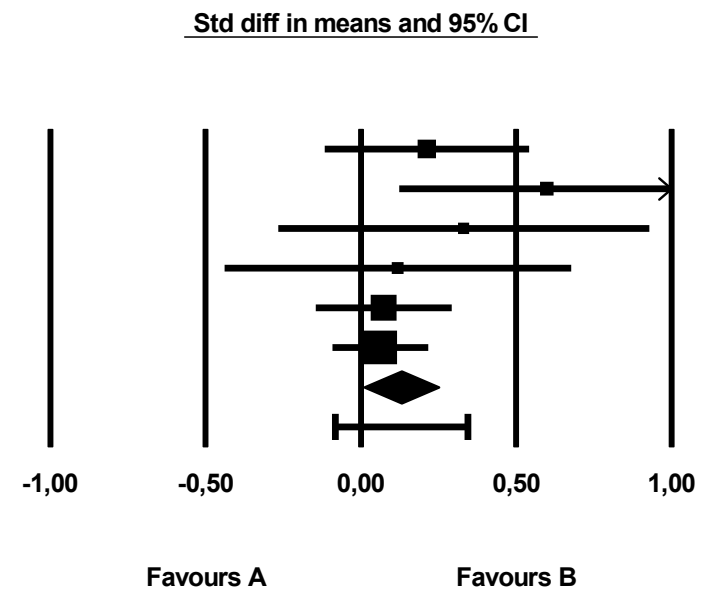

Meta Analysis

Figure S9. Forest plot of standardized mean differences in parent ratings of parental depression at post assessment across included studies. WL = waitlist control; NTC = no treatment control; Par-D = parental depression.
